# Integrative taxonomy reveals four new species of the armoured catfish genus *Pareiorhina* (Siluriformes: Loricariidae) from the upper Paraná River basin, Brazil

**DOI:** 10.1111/jfb.70319

**Published:** 2026-02-12

**Authors:** Pedro L. C. Uzeda, Luana J. Sartori, Axel M. Katz, Felipe P. Ottoni, Wilson J. E. M. Costa, Francisco Langeani, Valter M. Azevedo‐Santos

**Affiliations:** ^1^ Laboratório de Ecologia de Peixes, Departamento de Ecologia e Conservação Universidade Federal de Lavras Lavras Brazil; ^2^ Departamento de Biologia Universidade Federal de Lavras Lavras Brazil; ^3^ Laboratório de Sistemática e Evolução de Peixes Teleósteos, Instituto de Biologia Universidade Federal do Rio de Janeiro Rio de Janeiro Brazil; ^4^ Universidade Federal do Maranhão (UFMA), Centro de Ciências de Chapadinha, Laboratório de Sistemática e Ecologia de Organismos Aquáticos Chapadinha Brazil; ^5^ NRF‐South African Institute for Aquatic Biodiversity (NRF‐SAIAB) Makhanda South Africa; ^6^ Departamento de Ciências Biológicas UNESP – Universidade Estadual Paulista ‘Júlio de Mesquita Filho’ São José do Rio Preto Brazil; ^7^ Universidade Federal do Tocantins (UFT), Programa de Pós‐Graduação em Biodiversidade, Ecologia e Conservação Tocantins Brazil; ^8^ Grupo de Ecologia Aquática, Espaço Inovação do Parque de Ciência e Tecnologia Guamá (PCT Guamá) Belém Brazil; ^9^ Centro Universitário Eduvale São Paulo Brazil

**Keywords:** cryptic species, freshwater fishes, headwaters, Hypoptopomatinae, species delimitation

## Abstract

Four new species of the small‐sized armoured catfish genus *Pareiorhina* are described from mountain ranges in the Grande River drainage, upper Paraná River basin, based on morphological and molecular species delimitation methods. Molecular analyses based on the cytochrome oxidase subunit I (COI) marker recovered *Pareiorhina* as polyphyletic, with none of the 11 nominal species, including the 4 newly described, forming a clade with the type species *Pareiorhina rudolphi*. Instead, two sister clades were recovered outside *P. rudolphi*: one comprising species with a postdorsal ridge and unicuspid teeth, and another lacking the ridge and bearing bicuspid teeth. The four new species resemble *Pareiorhina carrancas* and *Pareiorhina hyptiorhachis*, both readily distinguished by the presence of a postdorsal ridge and simple teeth, and differ from all congeners by unique combinations of body colouration, abdominal plating, vertebral counts, pelvic girdle morphology and morphometric traits. These results underscore the underestimated diversity of the upper Paraná River basin and reinforce the need for continued ichthyofaunal surveys. Furthermore, they highlight the importance of integrating morphological and molecular data to unravel species diversity and distribution patterns of small‐sized fish species inhabiting southeastern Brazilian headwaters.

## 1 INTRODUCTION

*Pareiorhina* Gosline, 1947 comprehends a group of small suckermouth armoured catfishes along with over a hundred genera in the Neotropical family Loricariidae (Siluriformes) (Fricke et al., 2025). Currently, the genus is allocated in the tribe Neoplecostomini based on molecular data (Roxo et al., 2019).

Gosline (1947) proposed *Pareiorhina* to include a single species, *Rhinelepis rudolphi* Miranda Ribeiro, 1911. The genus remained monotypic for almost 60 years, until the description of *Pareiorhina carrancas* Bockmann & Ribeiro, 2003. In the same publication, Bockmann and Ribeiro (2003) compared morphological traits exhibited by species of *Pareiorhina* and taxa within the Neoplecostominae sensu Gosline (1947), and tentatively diagnosed *Pareiorhina* by the absence of an adipose fin, absence of hypertrophied odontodes on cheeks, absence of abdominal plates, absence of a lateral cusp in teeth, caudal peduncle distinctly rectangular in cross‐section, dorsal plates fused along the dorsal midline and ventral plates fused posteriorly to anal fin.

The only subsequent morphological assessment of *Pareiorhina* was carried out by Pereira and Reis (2017) in their morphology‐based phylogeny of the Neoplecostominae (=Neoplecostomini sensu Roxo et al., 2019), who recovered *Pareiorhina* as a monophyletic group based on 14 non‐exclusive synapomorphies. However, molecular analyses have repeatedly recovered *Pareiorhina* as a polyphyletic assemblage (Chiachio et al., 2008; Cramer et al., 2011; Roxo et al., 2019; Roxo, Albert, et al., 2014; Roxo, Silva, et al., 2014; Roxo, Zawadzki, et al., 2012). To this day, formal taxonomical arrangements reflecting phylogenetic relationships among nominal species of *Pareiorhina* supported by molecular data are still unavailable.

*Pareiorhina* is currently composed of seven nominal species distributed in three main drainages of the Brazilian Crystaline Shield, in southeastern Brazil. *Pareiorhina rudolphi* (Miranda Ribeiro 1911), *Pareiorhina brachyrhyncha* Chamon et al., 2005 and *Pareiorhina hyptiorhachis* Silva et al., 2013 are known from the Paraíba do Sul River basin (Chamon et al., 2005; Miranda Ribeiro, 1911; Silva et al., 2013). In turn, *Pareiorhina cepta* Roxo, Silva, et al., 2012 and *Pareiorhina rosai* Silva et al., 2016 occur in São Francisco River basin (Roxo, Silva, et al., 2012; Silva et al., 2016). Finally, *P. carrancas* and *Pareiorhina pelicicei* Azevedo‐Santos & Roxo, 2015 were described from the Grande River in the upper Paraná River basin (Azevedo‐Santos & Roxo, 2015; Bockmann & Ribeiro, 2003).

These seven species in the genus exhibit a wide range of morphological character states, which do not perfectly fit the definition provided by Bockmann and Ribeiro (2003) when compared to *P. rudolphi* (e.g., Roxo, Silva, et al., 2012). Among the species currently allocated in the genus, *P. carrancas* and *P. hyptiorhachis* are easily diagnosed by the presence of a postdorsal ridge, which is formed by a series of high azygous plates on the dorsal surface of the caudal peduncle (Bockmann & Ribeiro, 2003; Silva et al., 2013).

Throughout the past two decades, expeditions to headwater streams of the Grande River drainage in the upper Paraná River basin revealed several populations of *Pareiorhina* with morphological traits resembling *P. carrancas* and *P. hyptiorhachis* (i.e., Azevedo‐Santos et al., 2019; Casarim et al., 2012; Pompeu et al., 2009). Upon further examination, we observed that each series initially assigned to either of the two species exhibited unique character states or combinations, likely representing undescribed species. Our objectives here are to perform species delimitation analyses integrating morphological and molecular data and to provide formal descriptions of these new species.

## 2 MATERIALS AND METHODS

### 2.1 Species concept

We adopted the framework of the Unified Species Concept (De Queiroz, 2007), which defines species as independently evolving lineages, and can be identified by multiple lines of evidence. For morphological species delimitation, we applied the Population Aggregation Analysis (PAA), which identifies diagnostic groupings based on unique combinations of character states shared among individuals (Davis & Nixon, 1992). When available, we also considered the presence of derived character states that are unique to a single species (i.e., autapomorphies) (Rosen, 1979). For molecular species delimitation, we employed distance‐ and coalescent‐based approaches based on fragments of mitochondrial DNA gene cytochrome oxidase subunit I (COI) – see *Molecular species delimitation* section below. The congruence between morphological and molecular species was interpreted as additional support for the recognition of distinct evolutionary units.

### 2.2 Sampling

Samplings were conducted using sieves and dip‐nets. After capture, specimens were anaesthetized in eugenol solution (0.50 mL^−1^), photographed and preserved. Specimens designated for morphological analyses were fixed in 10% formalin for 7 days and later transferred to 70% ethanol for permanent storage and handling. Specimens intended for molecular analyses (listed as ‘DNA’ in paratypes lists) were placed in 99.2% ethanol and stored at −22°C for posterior tissue sampling.

### 2.3 Morphological analyses

#### 2.3.1 Morphological data

All measurements (following Bockmann & Ribeiro, 2003) were performed point‐to‐point on each individual to the nearest 0.1 mm, using a digital calliper under a stereomicroscope. Counts follow Bockmann and Ribeiro (2003), with the update provided by Silva et al. (2016) and were taken from the left side of the specimens. Measurements are presented as percentage of the standard length (SL) or head length (HL). Nomenclature of body plates and latero‐sensory canals follow Schaefer (1997) and Arratia and Huaquin (1995), respectively. Osteological examination and counts were taken from cleared and double‐stained (c&s) specimens prepared according to the method described in Taylor and Van Dyke (1985). Vertebral counts are presented as sum of Weberian apparatus (counted as five), intermediate vertebrae and compound caudal centrum (PU1 + U1) (counted as one). Osteological illustrations were made with a drawing tablet in Adobe Illustrator over pictures of dissected specimens. Institutional abbreviations follow Fricke and Eschmeyer (2025), with the addition of MZUFV (Ichthyological Collection of the Museu de Zoologia João Moojen, Universidade Federal de Viçosa, Viçosa, Brazil).

#### 2.3.2 Morphometric analysis

To explore overall morphological variation among *Pareiorhina* species bearing a postdorsal ridge within an arbitrary morphospace, we performed a principal component analysis (PCA) using the ‘PCA’ function from the FactoMineR package (Le et al., 2008) in R software (R Core Team, 2024). To minimize the effect of body size, all morphometric variables were expressed as proportions of SL or HL. To assess patterns of variation in count‐based traits, meristic data were also included, with the exception of the number of fin rays, which presented little or no variation. To ensure comparability across these traits, all variables (morphometric and meristic) were standardized using the z‐score transformation via the ‘scale’ function.

To further investigate interspecific differences in body shape and counts, we applied a linear discriminant analysis (LDA) using the ‘lda’ function from the MASS package (Venables & Ripley, 2002) on the standardized dataset. Finally, to statistically test for multivariate differences among species, we conducted a permutational multivariate analysis of variance (PERMANOVA) using the ‘adonis2’ function from the vegan package (Oksanen et al., 2022), with the standard 0.05 significance threshold. All analyses were performed using RStudio version 2024.04.1 (RStudio Team, 2024).

### 2.4 Molecular analyses

#### 2.4.1 DNA extraction, amplification and sequencing

Total genomic DNA was extracted from tissues from the right side of the caudal peduncle using the DNeasy Blood & Tissue Kit (Qiagen), following manufacturer's protocol. Fragments of mitochondrial DNA gene cytochrome c oxidase subunit 1 (COI) were amplificated using universal primers designed by Ward et al. (2005) (FISHF1 5´ TCAACCAACCACAAAGACATTGGCAC‐3′ and FISHR1 5´‐TAGACTTCTGGGTGGCCAAAGAATCA‐3′). For double‐stranded polymerase chain reaction (PCR) amplifications, we used 60 mL reactions with reagents at the following concentrations: 5 GreenGoTaq Reaction Buffer (Promega), 1 mm MgCl_2_, 1 mm of each primer, 75 ng of total genomic DNA, 0.2 mm of each dNTP and 1 U of Promega GoTaq Hot Start polymerase. The thermocycle profile was as follows: initial denaturation for 2 min at 95°C; 35 cycles of denaturation for 1 min at 94°C, annealing for 1 min at 50°C and extension for 1.3 min at 73°C. Negative controls were used to check for contaminations. Wizard SV Gel and PCR Clean Up System (Promega) was used in the PCR products for purification. Sanger sequencing reactions were made by SENAI SETIQ (Serviço Nacional de Aprendizagem Industrial) (Brazil). Cycle sequencing reactions were performed in 20 μL reaction volumes containing 4 μL BigDye, 2 μL sequencing buffer 5× (Applied Biosystems), 2 μL of the amplified products (10–40 ng), 2 μL primer and 10 μL deionized water. The thermocycling profile was (1) 35 cycles of 10 s at 96°C, 5 s at 54°C and 4 min at 60°C. MEGA11 (Tamura et al., 2021) was used for sequencing chromatograms and sequences.

#### 2.4.2 Taxon sampling and alignment

The dataset contained 14 COI sequences from topotypes of all of the seven valid *Pareiorhina* species, comprising one *P. rudolphi*, two *P. brachyrhyncha*, two *P. carrancas*, three *P. cepta*, three *P. pelicicei*, one *P. rosai* and two paratypes of *P. hyptiorhachis*. Also, we included 10 COI sequences belonging to four morphotypes to be tested here. We also included sequences of the neoplecostomins *Neoplecostomus microps* (Steindachner 1877), *Plesioptopoma curvidens* Reis et al., 2012, *Pseudotocinclus tietensis* (von Ihering, 1907) and the otothyrin *Schizolecis guntheri* (Miranda Ribeiro, 1918) as out‐groups. Sequences not generated for this project were obtained from the National Center for Biotechnology Information (NCBI) database. A list of specimens and their respective GenBank accession numbers is provided in Table S1. In MEGA11 (Tamura et al., 2021), sequences were aligned using the ClustalW algorithm (Chenna et al., 2003) and translated into protein sequences to check for stop codons or indels. We calculated the Kimura 2‐parameter (K2P) genetic distances between species in MEGA11.

#### 2.4.3 Molecular species delimitation

Four independent species delimitation methods based on molecular data were applied, each one with different operational criteria: The distance‐based method Assemble Species by Automatic Partitioning (ASAP), which identifies species boundaries by detecting significant gaps between the largest intraspecific and the smallest interspecific genetic distances (Puillandre et al., 2021); the coalescent‐based method Bayesian Poisson Tree Processes (bPTP), which infers species boundaries by modelling speciation and coalescent events as distinct Poisson processes and detecting shifts in substitution rates between intra‐ and interspecific branches (Zhang et al., 2013); the Generalized Mixed Yule Coalescent (GMYC) method, which detects the transition from interspecific speciation (Yule process) to intraspecific coalescence by analysing changes in branching rates over time; we implemented both the single‐threshold (sGMYC) and multiple‐threshold (mGMYC) models (Fujisawa & Barraclough, 2013). Finally, the Wiens and Penkrot (WP) tree‐based method identifies exclusive lineages in gene trees and assumes that their reciprocal monophyly reflects reproductive isolation, and thus potential species boundaries (Wiens & Penkrot, 2002).

The ASAP method was performed using the ASAP web server (https://bioinfo.mnhn.fr/abi/public/asap/asapold.html), where we directly uploaded the alignment file by applying the K2P nucleotide substitution model while keeping all other parameters at their default settings. For ASAP, the alignment file contained only sequences of our in‐group, and sequences from out‐groups (including *P. rudolphi*) were removed. We considered the partition with the lowest ASAP score as the optimal species delimitation.

For both the bPTP and GMYC methods, we first generated a reduced dataset containing only unique haplotypes using DAMBE5 (Xia, 2013) to avoid zero‐length branches. Given that our in‐group included *Pareiorhina* species that are distantly related to *P*. *rudolphi*, we excluded the type species from this dataset. Instead, we selected *P. curvidens* as the out‐group, as it was recovered as more closely related to our in‐group species in the phylogenetic hypothesis proposed by Roxo et al. (2019). From this reduced dataset, we inferred a Bayesian phylogenetic tree using BEAST version 1.10.4 (Suchard et al., 2018) under the following parameters: a strict molecular clock, the Birth–Death process as the tree prior, the TN + F + G4 substitution model (best‐fit model for this dataset), selected by the ModelFinder function (Kalyaanamoorthy et al., 2017) to calculate the best nucleotide substitution model and 30 million Markov chain Monte‐Carlo (MCMC) generations, with a tree sampling frequency of every 1000 generations. The maximum clade credibility tree was obtained using TreeAnnotator version 1.10.4 (Suchard et al., 2018) after discarding the first 10% of trees as burn‐in. The bPTP analysis was then conducted by uploading the resulting tree to the Exelixis Lab's PTP web server (https://species.h-its.org/ptp/), using default settings.

For the GMYC approach, an ultrametric tree was inferred in BEAST version 1.10.4 using an uncorrelated relaxed clock model with lognormal distribution, the Yule process as the tree prior, the TN + F + G4 substitution model and 30 million MCMC generations, with a tree sampling frequency of every 1000 generations. The maximum clade credibility tree was obtained using TreeAnnotator after a 10% burn‐in. This resulting tree was uploaded to the GMYC web server (https://species.h-its.org/gmyc/) under both single‐ and multiple‐threshold settings.

Finally, for the WP analysis, all sequences from in‐group and out‐groups were analysed in IQ‐TREE 1 (Nguyen et al., 2015) using the ModelFinder function (Kalyaanamoorthy et al., 2017) to calculate the best nucleotide substitution model. Bayesian inference (BI) and maximum likelihood (ML) analyses were performed using the software MrBayes 3.2 (Ronquist et al., 2012) and IQ‐Tree 1, respectively. The BI analysis was performed using the following parameters: two in‐dependent MCMC runs of two chains each for 30 million generations, with a tree sampling frequency of every 1000 generations. The convergence of the MCMC chains and the proper burn‐in value were assessed by evaluating the stationary phase of the chains using Tracer version 1.10.4 (Rambaut et al., 2018). The BI final consensus tree and its Bayesian posterior probabilities were generated with the remaining tree samples after the first 25% samples were removed as burn‐in. Methods used for assessing the reliability of internal branches in ML were ultrafast bootstrap support (UFBoot) (Minh et al., 2013); the Shimodaira‐Hasegawa‐like procedure support (SH‐aLRT) (Guindon et al., 2010); and the Bayesian‐like transformation of SH‐aLRT support (aBayes) (Anisimova et al., 2011) using 1000 replicates following default parameters implemented using IQ‐TREE 1.

#### 2.4.4 Molecular diagnosis (CBB)

The CBB is similar to the PAA proposed by Davis and Nixon (1992) but directed to nucleotides as an alternative method for diagnosing taxa through DNA barcodes (DeSalle et al., 2005; Hebert, Cywinska, et al., 2003; Hebert, Penton, et al., 2004; Hebert, Ratnasingham, & de Waard, 2003; Hebert, Stoeckle, et al., 2004). New species were diagnosed by the presence of a unique combination of nucleotide substitutions at particular sites, following Costa et al. (2014), Ottoni et al. (2019), Guimarães et al. (2020) and Aguiar et al. (2022). Nucleotide substitutions among lineages were optimized on the BI topology using PAUP version 4 (Swofford, 2002). Each nucleotide substitution is represented by its relative numeric position determined through sequence alignment with the complete mitochondrial COI gene of *Hypoptopoma incognitum* Aquino & Schaefer, 2010 (NC_028072.1: 5473‐7023), followed by the specific nucleotide substitution in parentheses.

### 2.5 Conservation status

We evaluated the conservation status of the species on the basis of the International Union for Conservation of Nature (IUCN) criteria (IUCN Standards and Petitions Subcommittee, 2024). We estimated the extent of occurrence (EOO) using QGIS software version 3.30.2 (QGIS, 2025) with the HydroBASINS database (Lehner & Grill, 2013), defining the minimum convex polygon as the total area of the microbasins encompassing the total known distribution of each species.

## 3 RESULTS

### 3.1 Morphology

**FIGURE 1:**
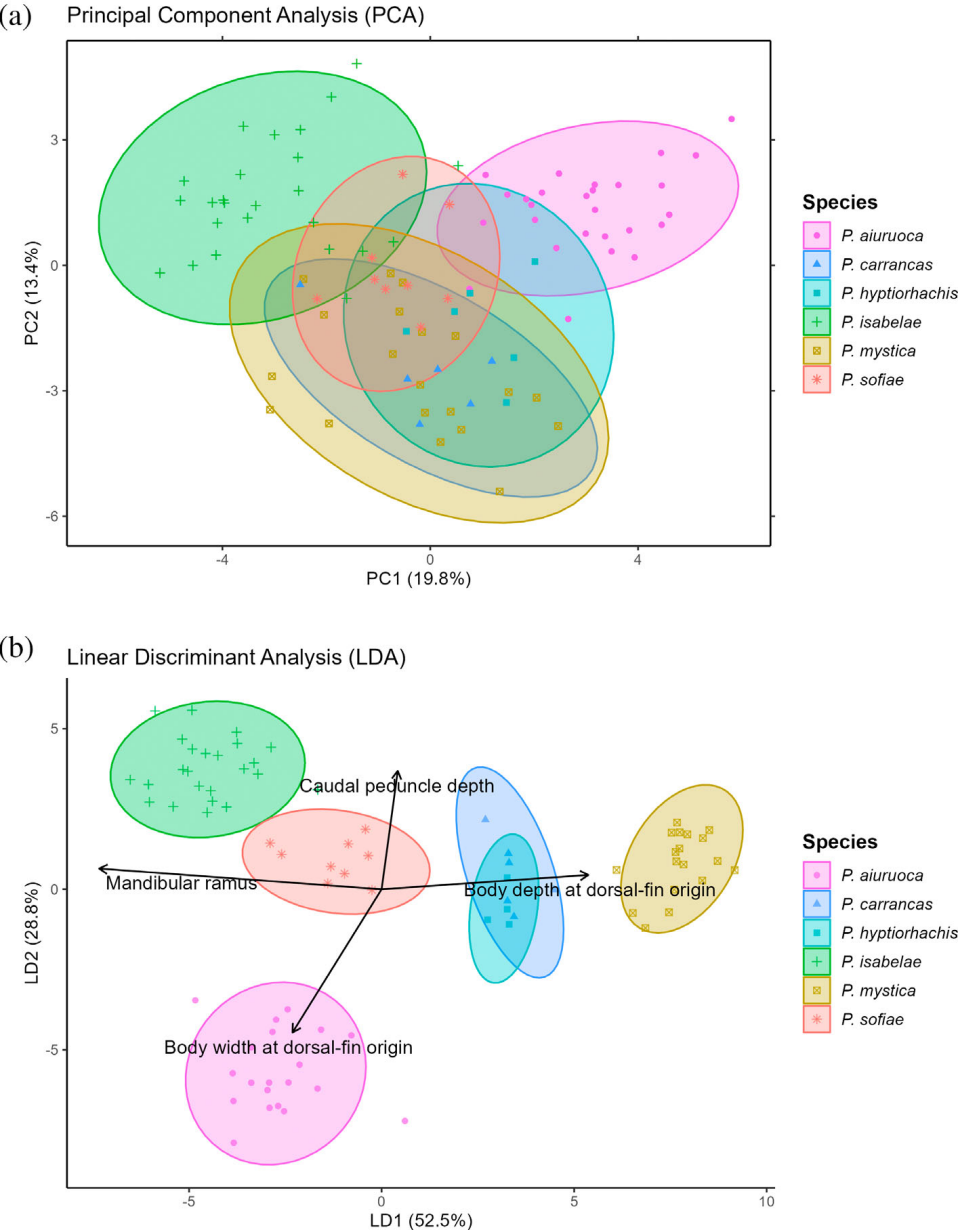
Biplots of morphometric analyses. (a) Principal component analysis. (b) Linear discriminant analysis. Ellipses represent 95% confidence intervals; vectors represent the most discriminant morphological traits [Correction added on 08 May 2026, after first online publication: The figure caption has been corrected in this version.].

The first two principal components (PC) accounted for 33.6% of the total morphological variation (19.8% in PC1 and 13.48% in PC2). The traits contributing most strongly to PC1 were the number of dentary teeth (loading = −0.213) and the number of plates in the mid‐dorsal series (loading = 0.305). For PC2, cleithral width (−0.367) and the number of plates between the dorsal and caudal fins (0.293) had the strongest contributions. Confidence interval ellipses revealed partial overlap among the six species analysed; however, *Pareiorhina aiuruoca* sp. n. showed no overlap with *P. carrancas* or *Pareiorhina isabelae* sp. n. (Figure 1a).

In the LDA, the first two discriminant functions explained 81.4% of the total interspecific variation (52.5% in LD1 and 28.8% in LD2). The most influential traits on LD1 were the width of the mandibular ramus (−2.799) and body depth at the dorsal‐fin origin (2.026). For LD2, caudal peduncle depth (−1.075) and body width at the dorsal‐fin origin (1.172) were the main contributors. The LDA effectively discriminated the species, with no overlap observed except between *P. carrancas* and *P. hyptiorhachis* (Figure 1b). This morphological differentiation was statistically supported using the PERMANOVA (*p* < 0.001), which supported all species as significantly distinct from one another, including *P. carrancas* and *P. hyptiorhachis* (Table 1). In addition to morpho‐meristic differentiation, all six species exhibited diagnostic characters in body colouration, abdominal plating and osteological features, supporting their recognition as distinct evolutionary lineages.

**TABLE 1:** Parameters of the global PERMANOVA model and pair‐wise permutational multivariate analysis of variance (PERMANOVA) between species.

PERMANOVA				
Degrees of freedom	Sum of squares	*R* ^2^	*F*	*p* (>F)
5	997.517265	0.328563	8.514568	0.001
87	2038.482735	0.671437	NA	NA
92	3036	1	NA	NA

Pair‐wise PERMANOVA					
Species 1	Species 2	*F*	*R* ^2^	*p*	Adjusted *p*
*P. aiuruoca*	*P. sofiae*	6.939982	0.169516	0.001	0.001154
*P. aiuruoca*	*P. carrancas*	7.093681	0.186217	0.001	0.001154
*P. aiuruoca*	*P. hyptiorhachis*	5.648822	0.154134	0.001	0.001154
*P. aiuruoca*	*P. isabelae*	20.16314	0.287375	0.001	0.001154
*P. aiuruoca*	*P. mystica*	11.1006	0.197869	0.001	0.001154
*P. sofiae*	*P. carrancas*	4.489635	0.256703	0.001	0.001154
*P. sofiae*	*P. hyptiorhachis*	4.396997	0.252745	0.001	0.001154
*P. sofiae*	*P. isabelae*	5.496344	0.146583	0.001	0.001154
*P. sofiae*	*P. mystica*	3.402198	0.111906	0.001	0.001154
*P. carrancas*	*P. hyptiorhachis*	4.747306	0.32191	0.002	0.002143
*P. carrancas*	*P. isabelae*	6.986702	0.194147	0.001	0.001154
*P. carrancas*	*P. mystica*	2.758959	0.103104	0.004	0.004
*P. hyptiorhachis*	*P. isabelae*	8.843332	0.233683	0.001	0.001154
*P. hyptiorhachis*	*P. mystica*	5.017837	0.172923	0.001	0.001154
*P. isabelae*	*P. mystica*	11.6036	0.212506	0.001	0.001154

*Note*: *p‐*Values < 0.05 are considered significant.

### 3.2 DNA barcoding, phylogenetic relationships and molecular species delimitation

The molecular dataset included sequences of 28 specimens, with a total length of 639 bp, of which 473 positions were conserved (166 variable) and 109 parsimony‐informative. Average nucleotide composition was 23.7% adenosine, 31.1% cytosine, 18.9% guanine and 26.3% thymine. Our sequences were considered to be suitable for phylogenetic analyses, as we found no saturation in our matrix (Iss < Iss.c S/Iss.c A). The overall average K2P genetic distance in our in‐group was 4.79%, ranging from 0.99% (*P. cepta* vs. *P. pelicicei*) to 8.52% (*P. brachyrhyncha* vs. *P. hyptiorhachis*) (Table 2).

**TABLE 2:** Genetic distances and standard deviation among *Pareiorhina* species, based on the Kimura 2‐parameter (K2P) model for the cytochrome oxidase subunit I (COI) marker.

	1	2	3	4	5	6	7	8	9	10
*1. P. rudolphi*										
*2. P. brachyrhyncha*	11.09 + 0.16									
*3. P. rosai*	10.93 + 0	2.63 + 0.14								
*4. P. cepta*	11.38 + 0.26	2.32 + 0.12	2.13 + 0							
*5. P. pelicicei*	11.62 + 0.13	2.77 + 0.13	2.33 + 0	0.99 + 0.07						
*6. P. carrancas*	10.05 + 0.16	8.49 + 0.17	7.64 + 0	7.11 + 0.24	7.3 + 0.15					
*7. P. aiuruoca*	9.51 + 0	7.15 + 0.11	6.21 + 0	6.04 + 0.05	6.4 + 0.1	1.37 + 0.09				
*8. P. mystica*	10.45 + 0.09	7.57 + 0.13	5.58 + 0.1	6.56 + 0.13	6.82 + 0.12	2.24 + 0.25	1.48 + 0.22			
*9. P. sofiae*	11.93 + 0	7.15 + 0.14	7.64 + 0	7.59 + 0.16	7.61 + 0.12	6.01 + 0.14	5.53 + 0	5.74 + 0.27		
*10. P. isabelae*	11.7 + 0	7.15 + 0.12	6.44 + 0	6.8 + 0.1	6.8 + 0.1	5.43 + 0.11	4.96 + 0	5.17 + 0.25	1.78 + 0	
*11. P. hyptiorhachis*	11.53 + 0	8.52 + 0.15	7.21 + 0	7.6 + 0.12	8.42 + 0	2.56 + 0	1.74 + 0	2.19 + 0.28	5.81 + 0	5.35 + 0

**FIGURE 2:**
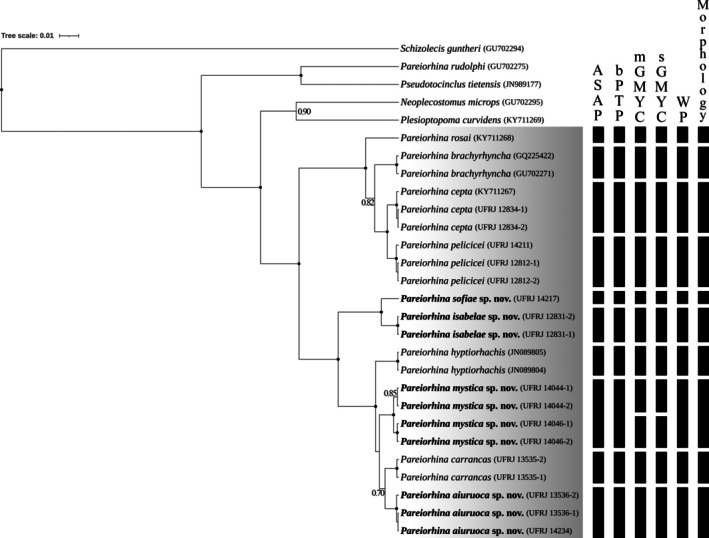
Bayesian Inference phylogenetic tree for the cytochrome oxidase subunit I (COI) marker. Black dots on nodes represent posterior probability values ≥0.99; values below 0.5 are not shown. Black bars at the right represent species delimited by the respective methods: Assemble Species by Automatic Partitioning (ASAP); Bayesian implementation of Poisson Tree process (bPTP); Generalized Mixed Yule‐Coalescent – multiple threshold (mGMYC); Generalized Mixed Yule‐Coalescent – single threshold (sGMYC); Wiens and Penkrot (WP); and morphology [Correction added on 08 May 2026, after first online publication: The figure caption has been corrected in this version.].

Our BI analysis recovered *Pareiorhina* as polyphyletic, comprising two distinct lineages: *Pareiorhina* sensu stricto, represented solely by the type species *P. rudolphi*, which was recovered as sister to *P. tietensis*; and a larger clade containing all remaining species, recovered as a sister clade to *N. microps* + *P. curvidens* (Figure 2). Within this larger clade, two well‐supported subclades were identified: the *P. carrancas* clade, comprising *P. carrancas*, *P. hyptiorhachis* and four new species; and the *P. brachyrhyncha* clade, comprising *P. brachyrhyncha*, *P. cepta*, *P. pelicicei* and *P. rosai*. Both the polyphyly of *Pareiorhina* and the monophyly of the *P. carrancas* and *P. brachyrhyncha* clades are supported by maximum Bayesian posterior probabilities (Figure 2).

The ASAP, bPTP and WP methods equally delimited 10 lineages: *P. brachyrhyncha*, *P. cepta*, *P. pelicicei*, *P. rosai*, *P. carrancas*, *P. hyptiorhachis*, *P. aiuruoca* sp. n., *P. isabelae* sp. n., *Pareiorhina mystica* sp. n. and *Pareiorhina sofiae* sp. n. The sGMYC and mGMYC methods equally delimited 11 lineages: *P. brachyrhyncha*, *P. cepta*, *P. pelicicei*, *P. rosai*, *P. carrancas*, *P. hyptiorhachis*, *P. aiuruoca* sp. n., *P. isabelae* sp. n., *P. sofiae* sp. n. and two lineages within *P. mystica* sp. n. Given that all morphological species are corroborated by all molecular species delimitation methods, we follow the consensus and describe four new species of *Pareiorhina* below.

## 4 TAXONOMIC ACCOUNTS

### 4.1 ***P. aiuruoca*, sp. n**. Uzeda, Katz, Ottoni, Costa, Langeani & Azevedo‐Santos

**FIGURE 3:**
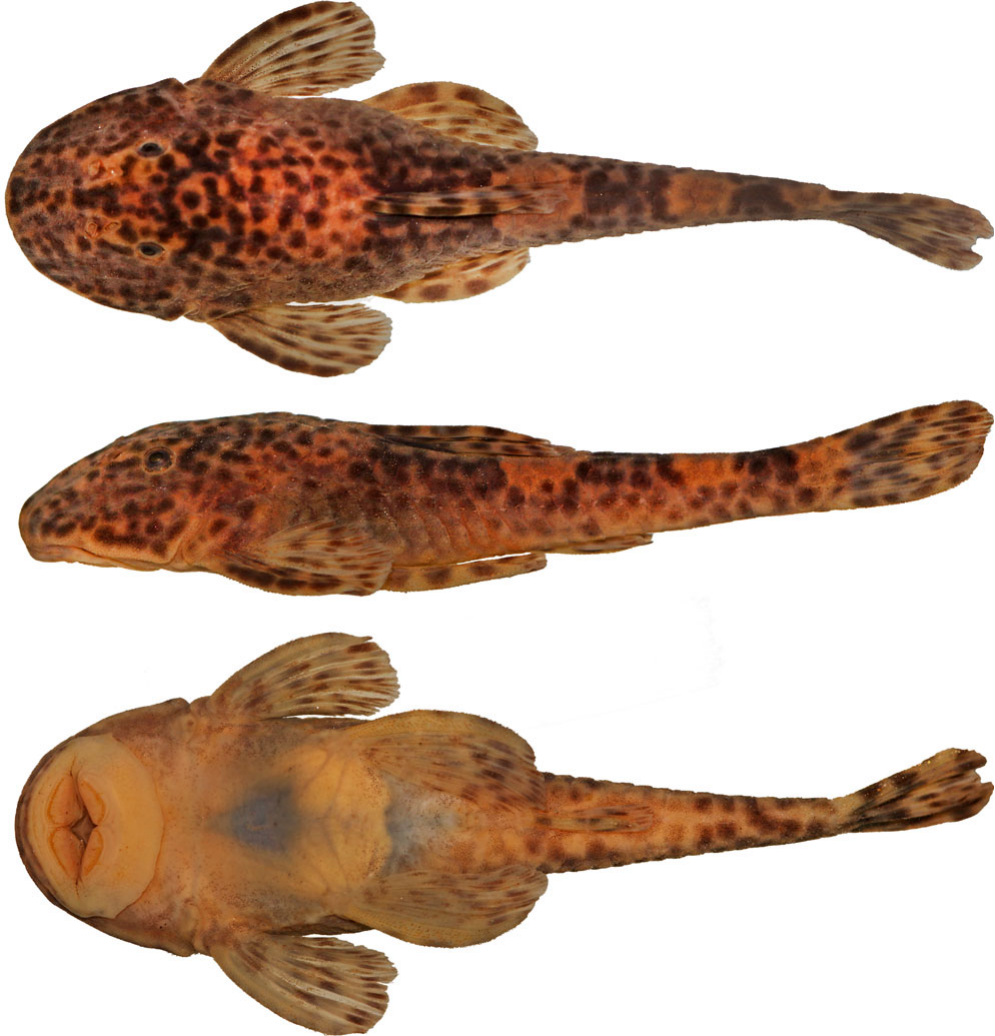
*Pareiorhina aiuruoca* **sp. n**., CI‐UFLA 2359, male, 42.2 mm standard length (SL), Brazil, Minas Gerais, Alagoa, Córrego do Quilombo, affluent to Aiuruoca River. Dorsal, lateral and ventral views.

urn:lsid:zoobank.org:act:B7FE1CF2‐64B7‐4380‐A53A‐8C253F0C51F9

(Figures 3 and 7a,b; Table 3)

**TABLE 3:** Morphometric and meristic data of *Pareiorhina aiuruoca* **sp. n**. and *Pareiorhina isabelae* **sp. n**.

Species	*Pareiorhina aiuruoca*, sp. nov. (*n* = 27)				*Pareiorhina isabelae*, sp. nov. (*n* = 25)			
	Holotype	Range	Mean	SD	Holotype	Range	Mean	SD
Standard length	42.2	28.6–42.2	35.5	2.7	36.4	28.6–38.6	33.7	2.6
Percentage of standard length								
Head length	13.3	31.2–36.1	33.1	1.2	11.9	30.2–42.6	32.6	2.4
Predorsal length	17.5	41.4–47.3	44.7	1.4	15.6	42.2–45.5	43.7	1.0
Pre‐anal length	25.6	58.2–63.7	61.6	1.2	21.6	56.5–61.5	58.8	1.1
Dorsal‐fin spine length	9.1	18.3–22.7	21.1	1.1	6.7	17.6–21.7	20.0	1.0
Dorsal‐fin base length	5.7	12.6–16.5	14.1	1.1	4.4	11.7–14.9	13.0	0.8
Anal‐fin spine length	6.2	11.0–16.9	15.1	1.1	5.0	13.1–16.5	14.5	0.8
Pectoral‐fin spine length	8.0	18.9–25.7	21.0	1.3	6.4	16.5–20.3	18.6	1.1
Lower caudal‐fin ray length	8.1	18.5–26.0	22.8	2.1	8.5	21.1–28.0	25.3	1.9
Trunk length	7.3	13.8–17.9	15.9	1.0	4.7	12.1–17.4	14.1	1.2
Abdominal length	9.3	19.7–25.3	23.3	1.4	8.2	21.0–25.4	23.2	1.0
Pelvic‐fin spine length	9.5	19.5–26.0	22.3	1.9	8.7	18.7–25.6	22.0	1.9
Cleithral width	13.0	30.7–33.8	32.4	0.9	11.2	29.5–33.4	31.5	1.0
Body depth at dorsal‐fin origin	7.2	16.7–21.8	19.1	1.6	5.9	15.4–19.7	17.1	1.1
Body width at dorsal‐fin origin	10.1	23.9–33.2	27.4	2.6	8.2	21.1–27.3	23.3	1.6
Body width at anal‐fin origin	5.1	12.1–14.8	13.3	0.6	4.5	11.5–14.2	12.8	0.7
Caudal peduncle depth	3.4	7.4–16.8	8.5	1.7	2.7	7.3–9.5	8.5	0.7
Percentage of head length								
Head width	12.9	88.6–100.4	95.1	3.5	11.2	72.1–103.0	95.1	5.6
Snout length	8.0	52.3–62.3	58.8	2.2	7.7	42.9–64.9	60.6	4.2
Orbital diameter	1.6	9.8–13.5	11.5	1.0	1.4	9.9–13.5	11.9	1.0
Interorbital width	4.4	33.3–40.4	36.8	1.8	4.1	24.5–36.8	34.1	2.3
Head depth	6.7	50.3–66.7	55.4	4.2	5.6	38.5–56.4	51.5	4.0
Suborbital depth	4.4	29.9–35.9	33.2	1.6	4.4	24.9–36.5	33.4	2.5
Mandibular ramus	2.7	14.2–20.4	18.1	1.6	2.8	15.6–26.1	21.5	2.1
Counts			Mode				Mode	
Dorsal plates	26	25–27	26	0.8	24	24–26	24	0.8
Mid‐dorsal plates	21	18–22	19	1.2	18	13–19	16	1.7
Median plates	26	25–28	26	0.7	25	24–27	25	1.0
Mid‐ventral plates	20	19–27	20	1.9	20	15–20	17	1.1
Ventral plates	21	21–24	22	0.9	21	21–23	21	0.7
Plates between dorsal and caudal fins	15	15–16	15	0.5	15	14–16	15	0.7
Plates between anal and caudal fins	12	12–14	13	0.6	13	12–14	13	0.6
Plates at dorsal‐fin base	5	5–6	6	0.5	5	4–6	5	0.6
Plates at anal‐fin base	2	2–3	2	0.4	3	2–3	2	0.4
Postdorsal ridge plates	11	9–14	13	1.5	13	11–16	12	1.3
Dorsal‐fin branched rays	7	7–7	7	0.0	7	6–7	7	0.2
Pectoral‐fin branched rays	6	6–6	6	0.0	6	6–6	6	0.0
Pelvic‐fin branched rays	5	5–5	5	0.0	5	5–5	5	0.0
Anal‐fin branched rays	5	5–5	5	0.0	5	5–5	5	0.0
Caudal‐fin branched rays	13	12–14	13	0.7	14	14–14	14	0.0
Premaxillary teeth	35	34–54	47	4.9	57	47–73	57	5.9
Dentary teeth	33	33–52	44	4.5	53	42–78	53	7.8

Abbreviation: SD, standard deviation.

‘*P. carrancas*’ – Casarim et al., 2012: p. 1171 [ichthyofauna inventory]

‘*P. carrancas*’ – Silva‐Sene et al., 2024: p. 4 [*p.p*., ichthyofauna inventory]

‘*P. carrancas’* – Dagosta et al., 2024: p. 41, fig. 21E [*p.p*., distribution map]

#### 4.1.1 Holotype

CI‐UFLA 2359, 42.2 mm SL, male, Córrego do Quilombo, tributary of Aiuruoca River drainage, Grande River, upper Paraná basin. Municipality of Alagoa, Minas Gerais, Brazil, 22°11′34″S 44°41′50″W, 1274 m a.s.l., 07 April 2023, P.L.C. Uzeda and L.J. Sartori.

#### 4.1.2 Paratypes

All from Aiuruoca River drainage, Minas Gerais, Brazil. **Córrego do Quilombo:** CI‐UFLA 1697, 11 specimens, 7 females (33.4–36.3 mm SL), 4 males (36.5–38.2 mm SL), 01 April 2010, R. Casarim, I.G Prado et al. CI‐UFLA 1698, 14 specimens, 7 females (31.5–37.6 mm SL), 4 males (35.2–36.5 mm SL), 3 unsexed (28.6–23.0 mm SL), collected with holotype. CI‐UFLA 2415, 4 c&s specimens, 34.1–36.6 mm SL, 01 April 2010, R. Casarim, I.G Prado et al. DZSJRP 23407, 6 specimens, 4 females (34.0–37.0 mm SL; 1, 35 mm SL, c&s), 2 males (36.54–37.3 mm SL; 1, 35.2 mm SL, c&s), 01 April 2010, R. Casarim, I.G. Prado et al. UFRJ 14150, 5 specimens, 2 females (34.3–35.6 mm SL), 3 males (36.0–37.7 mm SL), 01 April 2010, R. Casarim, I.G Prado et al. UFRJ 13536, 4 DNA, 30.7–39.6 mm SL, collected with holotype. UFRJ 14231, 10 specimens, 8 females, 2 males (37.0–40.4 mm SL), collected with holotype. **Córrego Cangalha:** CI‐UFLA 1849, 26 specimens, 13 females (27.3–36.5 mm SL), 8 males (30.7–40.6 mm SL), 5 unsexed (16.3–24.7 mm SL). Municipality of Aiuruoca at 22°5′26″S 44°36′59″W, 1145 m a.s.l., 11 August 2023, P.L.C. Uzeda and L.J. Sartori. DZSJRP 24844, 8 males (31.8–38.9 mm SL), 11 August 2023, P.L.C. Uzeda and L.J. Sartori. MZUFV 13604, 7 specimens, 3 females (34.1–34.5 mm SL), 4 males (36.3–37.6 mm SL), 11 August 2023, P.L.C. Uzeda and L.J. Sartori. UFMG‐ICT 4020, 11 specimens, 4 females (29.7–34.0 mm SL), 7 males (32.8–39.5 mm SL), 11 August 2023, P.L.C. Uzeda and L.J. Sartori. UFRJ 14244, DNA, 1 specimen, 37.0 mm SL, 11 August 2023, P.L.C. Uzeda and L.J. Sartori. **Córrego da Olaria**: CI‐UFLA 3763, 9 specimens, 5 females (37.6–44.3 mm SL), 3 males (39.2–41.8 mm SL), 1 unsexed (19.7 mm SL). Municipality of Aiuruoca at 22°8′14″S 44°37′11″W, 1114 m a.s.l., 01 May 2010, R. Casarim, I.G. Prado et al. CI‐UFLA 3764, 3 c&s specimens, 38.1–41.5 mm SL, 01 May 2010, R. Casarim, I.G Prado et al. UFRJ 14234, DNA, 2 females, 36.8–41.6 mm SL, 11 August 2023, P.L.C. Uzeda and L.J. Sartori. **Unnamed stream:** CICCAA 08619, 14 specimens, 7 females (36.5–38.9 mm SL), 7 males (38.8–43.6 mm SL). Municipality of Itamonte at 22°18′1”S 44°42′4”W, 1403 m a.s.l., 16 October 2010, R. Casarim, I.G. Prado et al.

#### 4.1.3 Non‐type specimens

**Ribeirão do Papagaio**: CI‐UFLA 1851, 20 specimens, 4 females (33.5–40.7 mm SL), 2 males (42.2–42.5 mm SL), 14 unsexed (19.9–26.2 mm SL). Municipality of Aiuruoca at 22°0′40″S 44°36′43″W, 1034 m a.s.l., 07 April 2023, P.L.C. Uzeda and L.J. Sartori. UFRJ 14036, DNA, 2 females, 33.3–33.7 mm SL, 07 April 2023, P.L.C. Uzeda and L.J. Sartori. **Córrego do Campo Redondo:** CI‐UFLA 3765, 2 specimens, 1 male (39.1 mm SL), 1 female (41.0 mm SL). Municipality of Itamonte at 22°15′60″S 44°41′42″W, 1553 m a.s.l., 06 August 2010, R. Casarim, I.G. Prado et al. **Córrego do Meio:** CI‐UFLA 3766, 5 specimens, 3 males (41.2–42.5 mm SL), 2 females (37.7–37.0 mm SL). Municipality of Itamonte at 22°14′35″S 44°42′40″W, 1375 m a.s.l., 31 March 2010, R. Casarim, I.G Prado et al. **Ribeirão da Berta:** CI‐UFLA 3767, 9 specimens, 5 males (35.1–37.5 mm SL), 4 females (34.0–37.8 mm SL). Municipality of Itamonte at 22°14′34″S 44°43′37″W, 1430 m a.s.l., 31 March 2010, R. Casarim, I.G. Prado et al.

#### 4.1.4 Diagnosis

**FIGURE 4:**
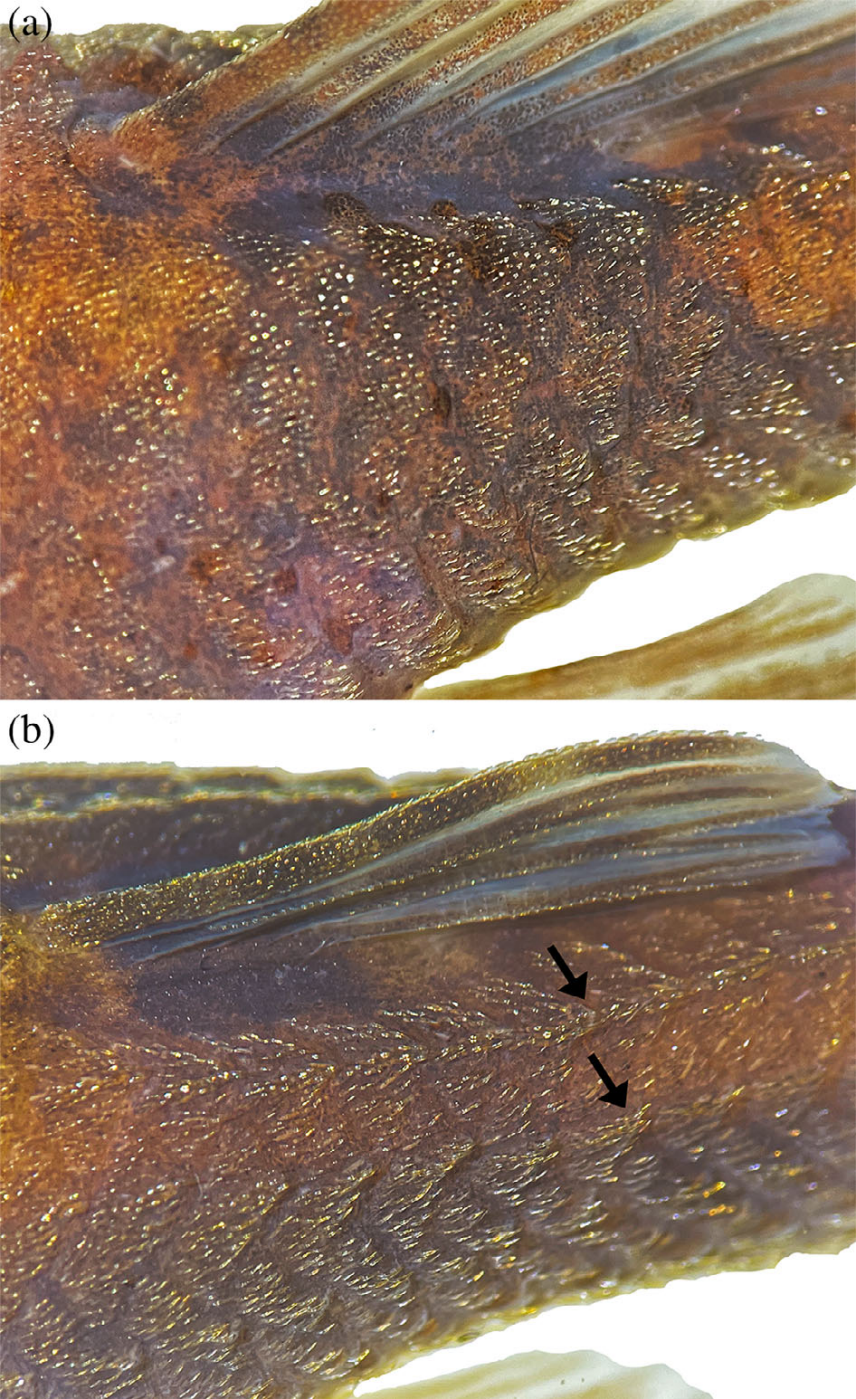
Left view of lateral series of plates. (a) *Pareiorhina aiuruoca* **sp. n**., paratype, MZUFV 13604, 37.0 mm SL, showing the absence of keels on lateral series of plates. (b) *Pareiorhina carrancas*, CI‐UFLA 1510, 35.3 mm SL, showing the moderate keels on dorsal and mid‐dorsal series of plates, formed by slightly hypertrophied and aligned odontodes.

**FIGURE 5:**
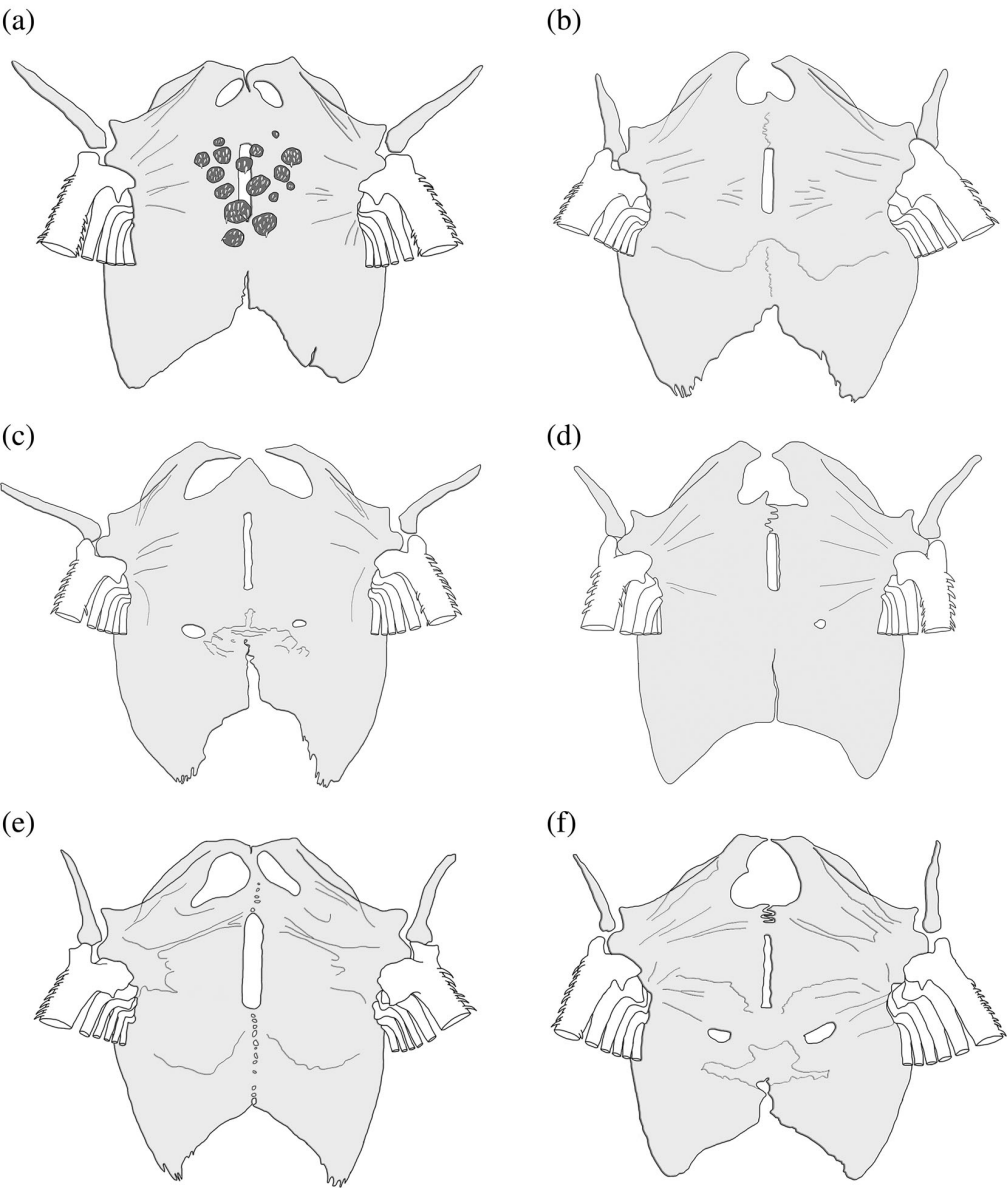
Pelvic girdle of species of the *Pareiorhina carrancas* clade, ventral view. (a) *Pareiorhina aiuruoca* **sp. n**., c&s, paratype, CI‐UFLA 2415. (b) *Pareiorhina isabelae* **sp. n**., c&s, paratype, CI‐UFLA 2333. (c) *Pareiorhina mystica* **sp. n**., c&s, paratype, CI‐UFLA 3761. (d) *Pareiorhina sofiae* **sp. n**., c&s, paratype, CI‐UFLA 3757. (e) *Pareiorhina hyptiorhachis*, c&s, paratype, LBP 12248. (f) *P. carrancas*, c&s, CI‐UFLA 3762.

*P. aiuruoca* can be readily diagnosed from all congeners by having a cluster of abdominal platelets between the pelvic fins (Figure 5a) (vs. abdomen naked or platelets randomly scattered over the abdominal surface when present). Additionally, *P. aiuruoca* differs from *P. rudolphi*, *P. brachyrhyncha*, *P. cepta*, *P. pelicicei* and *P. rosai* by the presence of a postdorsal ridge (vs. postdorsal ridge absent) and, except for *P. rudolphi*, by having unicuspid teeth (vs. bicuspid teeth). Among congeners with a postdorsal ridge, *P. aiuruoca* further differs from *P. carrancas* by the absence of keels on dorsal and mid‐dorsal series of plates (Figure 4a) (vs. presence, Figure 4b), by having two fenestrae on the anterior portion of pelvic girdle (Figure 5a) (vs. a single fenestra, Figure 5f) and 30 total vertebrae (vs. 31); and from *P. hyptiorhachis* by presenting dorsal profile of the snout elliptical (vs. rounded), snout tip naked (vs. covered with odontodes) and 30 total vertebrae (vs. 29).

**FIGURE 6:**
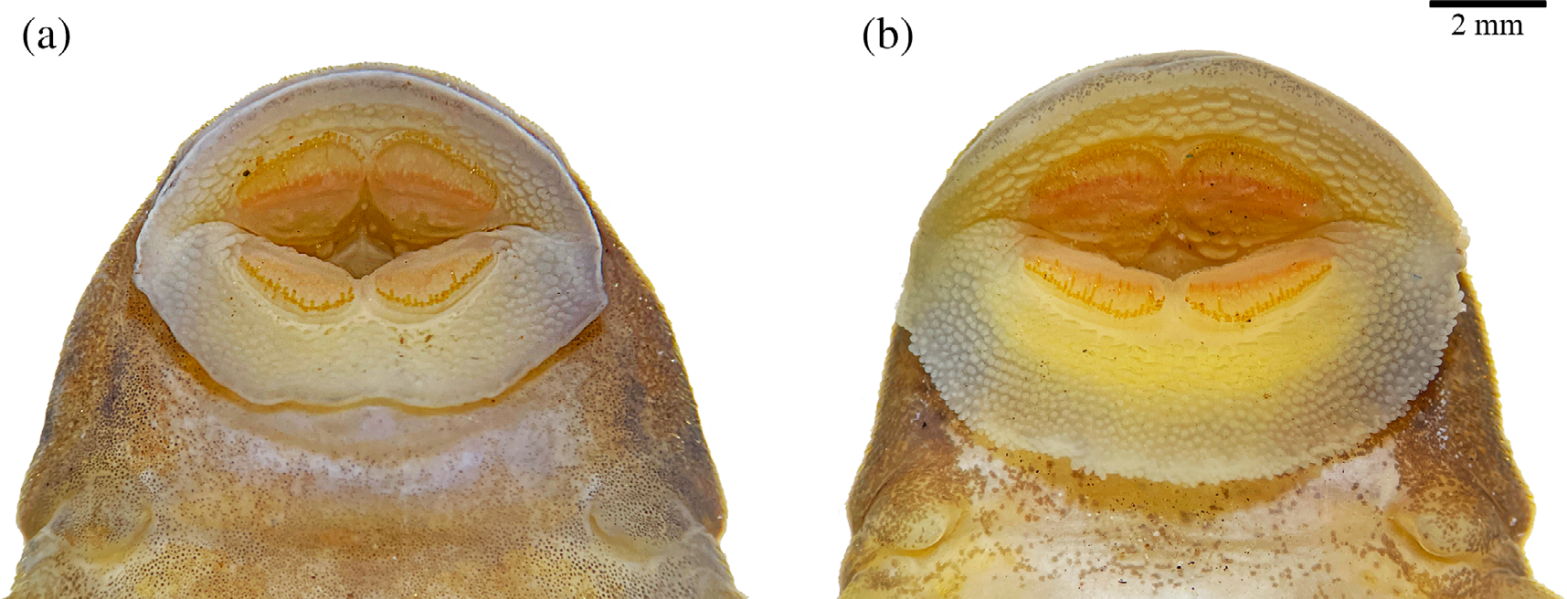
Ventral view of *Pareiorhina* mouths. (a) *Pareiorhina aiuruoca*, paratype, MZUFV 13604, with posterior margin of lower lip smooth. (b) *Pareiorhina isabelae*, paratype, MZUFV 13605, with posterior margin of lower lip fringed, ornamented with minute papillary projections.

Among the new congeners with a postdorsal ridge, *P. aiuruoca* can be further distinguished from *P. isabelae* **sp. n**., *P. mystica* **sp. n**. and *P. sofiae* **sp. n**. by the presence of dark spots over dorsal and lateral surfaces of head, body and fins (vs. head, body and fins homogeneously light cream or brown); and by having the margin of the lower lip smooth (Figure 6a) (vs. margin of lower lip fringed, Figure 6b). It further differs from *P. isabelae* **sp. n**. by normally having more plates on mid‐dorsal (18–22, modally 19 vs. 13–19, modally 16) and mid‐ventral series (19–27, modally 20 vs. 15–20, modally 17), two fenestrae on the anterior portion of pelvic girdle (vs. a single fenestra, Figure 5b) and 30 total vertebrae (vs. 31 vertebrae); from *P. mystica* **sp. n**. by the presence of azygous plates on predorsal region (vs. absence); and from *P. sofiae* **sp. n**. by having a longer pectoral‐fin spine (18.9%–25.7% SL vs. 16.0%–18.8%), reaching the second third of dorsal‐fin base when adpressed (vs. tip of pectoral‐fin spine reaching up to dorsal‐fin spine when adpressed, normally reaching nuchal plate), by having five infraorbitals (vs. four), snout tip naked (vs. covered by platelets), two fenestrae on the anterior portion of pelvic girdle (vs. a single fenestra, Figure 5d) and 30 vertebrae (vs. 29 vertebrae).

#### 4.1.5 Remarks

*P. aiuruoca* is the only species in the genus to present variation in the number of branched caudal‐fin rays in all analysed specimens and type series, ranging from 12(6) to 14(8), modally 13(13) branched rays. Although some specimens exhibit 14 branched caudal‐fin rays, a character state broadly distributed among the Loricariidae, the variation observed in *P. aiuruoca* is unique in *Pareiorhina* and can be used as an additional diagnostic character for the species.

#### 4.1.6 Molecular diagnosis (CBB)

*P. aiuruoca* is diagnosed molecularly by a combination of four nucleotide substitutions (listed below):

COI 477 (A → G), COI 513 (A → G), COI 568 (G → A), COI 672 (A → G).

#### 4.1.7 Description

**FIGURE 7:**
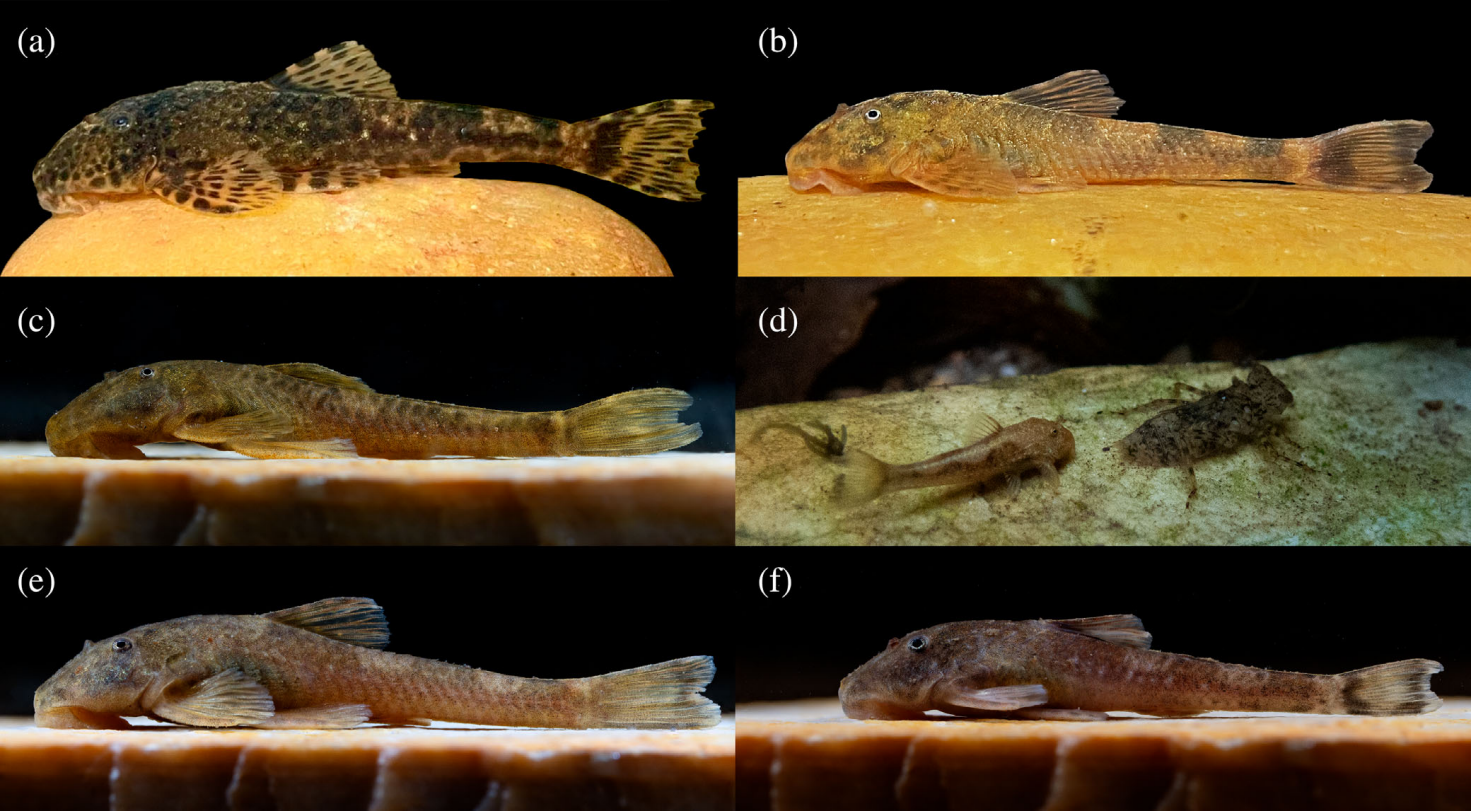
Live colouration of the new *Pareiorhina* species; adults to left and juveniles to right. (a) *Pareiorhina aiuruoca* **sp. n**., CI‐UFLA 1851. (b) *P. aiuruoca* sp. n., paratype, CI‐UFLA 1849. (c) *Pareiorhina isabelae* **sp. n**., paratype, UFRJ 12831. (d) Young specimen of *P. isabelae* **sp. n**. (not captured) near an Odonata nymph at the type locality. (e, f) *Pareiorhina mystica* **sp. n**., paratypes, UFRJ 14532.

Morphometric data and counts are presented in Table 3. Overall body shape and colouration in dorsal, lateral and ventral views are illustrated in Figures 3, 6a, and 7a,b. Head deep and snout short. Snout tip with small oval naked area. Nares located on first third of HL. Eyes small, with diameter similar to nares; dorsolaterally located, approximately on middle of HL. Iris diverticulum present and poorly developed. Interorbital region about four times eye diameter. Parieto‐supraoccipital higher than sphenotic, posteriorly bordered by two to four plates. Cheeks slightly above half of head depth; hypertrophied odontodes absent. Mouth broad and sucker shaped. Lips broad and covered with minute rounded papillae; posterior margin of lower lip smooth, without fringe or emarginations, almost reaching branchial slit transversally. Maxillary barbel short and adnate to lower lip, without visible tip. Mandibular rami broad, with minute rounded papillae immediately behind tooth series. Dentaries angled towards each other about 160°. Teeth simple, without lateral cusp; crown elongated and bronze coloured, with 34 to 54 premaxillary teeth and 33 to 52 dentary teeth.

Body covered with dermal plates, except for ventral surfaces of head, abdomen, the region overlying opening of swim‐bladder capsule and around bases of dorsal, pectoral, pelvic and anal fins. Presence of platelets covered with odontodes clustered over lateral portion of pectoral girdle, posterior to branchiostegal membrane and in area between pelvic fins. Abdominal platelets about pupil diameter, normally 10–20 clustered between pelvic fins, each one supporting over 10 odontodes in adult specimens. Dermal plates homogeneously covered with odontodes; plates diffuse in juvenile specimens. Plate series above lateral line roughly rounded or squared, ventral series vertically elongated. Dorsal series with 25–27 plates; mid‐dorsal series truncated with 18–22 plates, ending slightly posterior to end of dorsal‐fin base; median series with 25–28 plates; mid‐ventral series truncated with 19–27 plates, ending approximately at half the length of caudal peduncle; ventral series with 21–24 plates. Lateral line incomplete, ending at two to four posteriormost median plates. Dorsal surface of caudal peduncle with one series of 9–14 juxtaposed azygous plates, from tip of adpressed dorsal fin to procurrent caudal‐fin rays.

Dorsal fin ii,7, originating at vertical through median pelvic‐fin rays, slightly surpassing anal‐fin base when adpressed. Dorsal‐fin basal radials lying above neural spines of the 7th–14th vertebrae. Dorsal‐fin spinelet roughly rectangular; locking mechanism not functional. Pectoral fin i,6, reaching first half of pelvic fin and first third of dorsal fin in vertical when adpressed; presence of dark spots over rays, forming four or five diffuse transversal bands. Pelvic fin i,5, originating slightly anterior to dorsal‐fin origin in vertical; posterior margin reaching halfway between anal opening and anal‐fin origin in females, and surpassing anal‐fin origin in males. Unbranched ray curved inwards, its ventral surface covered with depressed, pointed and inward‐directed odontodes. Anal fin i,5, originating slightly posterior to end of dorsal‐fin base, reaching about half length of caudal peduncle when adpressed. Anal‐fin basal radials lying below haemal spines of the 14th–17th vertebrae. Caudal fin bilobed, with 14–16 principal rays, modally 15; upper lobe with I + 6–7, lower lobe with 6–7 + I. Two dorsal and two ventral procurrent rays, respectively, supported by neural and haemal spines of the 29th vertebral centrum. Total vertebrae 30; 14 precaudal, 16 caudal. Six or seven pleural ribs.

Supraorbital sensory canal with four pores; pore s1 located anteromesially to nare, pore s3 located posteriomesially to nare; pore s6 + s6 located in middle of interorbital region, pore s8 located posteromesially to eye, longitudinally aligned to mesial margin of nare. Infraorbital sensory canal with six pores; pore io1 located at middle of inferior margin of first infraorbital; pore io2 located between first and second infraorbitals; pore io3 located between second and third infraorbitals; pore io4 located between third and fourth infraorbitals; pore io5 located between fourth and fifth infraorbitals and pore io6 located between superior margin of fifth infraorbital, inferior margin of sphenotic and anterior margin of compound pterotic. Preoperculomandibular canal with four pores; pore pm1 located between posterior margin of cheek plate and anterior margin of subocular cheek plate; pore pm2 located in lateral region of cheek plate; pore pm3 located between dorsal margin of cheek plate and ventral margin of preopercle; pore pm4 located between preopercle and compound pterotic. Two postotic pores; pore po2 located above branchial slit and pore po3 located on lower half of compound pterotic.

#### 4.1.8 Colouration

Live specimens with dorsal colouration dark brown to dark orange with four light saddles on dorsum, located at dorsal‐fin origin, dorsal‐fin end, at half of caudal peduncle length and caudal peduncle end. Dark spots over brownish background on dorsal surface of head, body and fins, occasionally forming vermiculate pattern, mainly on head. Dark spots dispersed over fin rays, occasionally forming four or five diffuse bands; interradial membranes transparent brown (Figure 7a,b). In ventral view, light‐beige colouration from mouth to urogenital region, with melanophores concentrated near plated areas. Dark brown pigmentation on lateral surfaces of head and trunk, becoming predominant from urogenital region to end of caudal fin. Colouration in alcohol similar to that in life, but with fainted tones. Dorsal bars and spots become less conspicuous in most specimens, and interradial membranes become dim.

#### 4.1.9 Sexual dimorphism

Mature males have a conspicuous, conical urogenital papilla just posterior to anal opening (absent in females) and a fleshy flap on dorsal surface of pelvic‐fin spine (absent in females). Pelvic fins slightly surpassing anal‐fin origin in males, and not reaching anal fin in females. Reproductive females present a strong enlargement in body width between dorsal‐fin origin and anal‐fin origin, resulting in a bulging trunk in dorsal view (enlargement absent in males).

#### 4.1.10 Distribution

**FIGURE 8:**
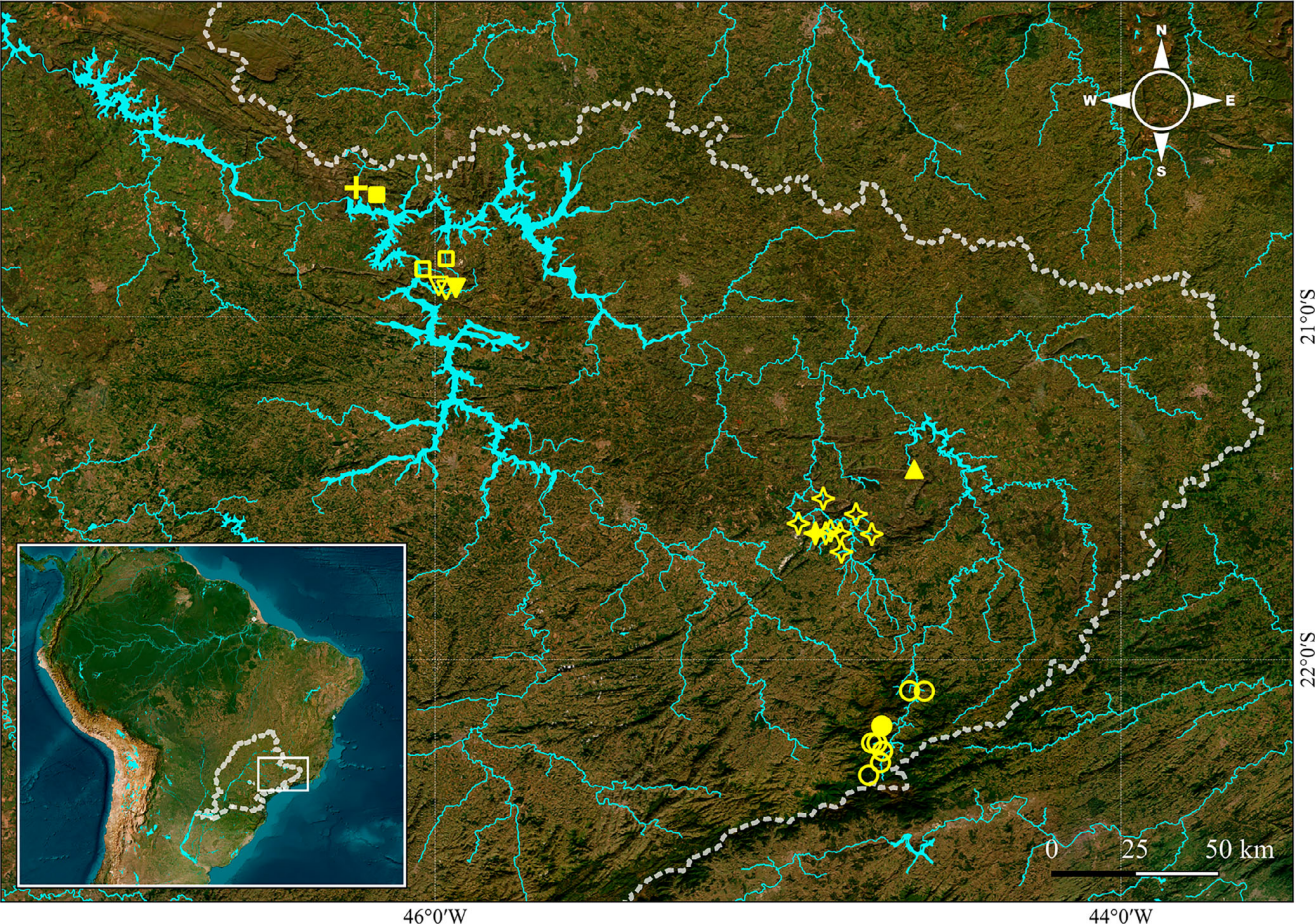
Distribution map of *Pareiorhina* species in the upper Grande River, upper Paraná River basin. Species are represented as follows: circles = *Pareiorhina aiuruoca* **sp. n**.; cross = *Pareiorhina sofiae* **sp. n**.; inverted triangles = *Pareiorhina isabelae* **sp. n**.; squares = *Pareiorhina pelicicei*; stars = *Pareiorhina mystica* **sp. n**.; triangle = *Pareiorhina carrancas*. The white dashed line delimits the upper Paraná River basin; full symbols represent type localities.

*P. aiuruoca* is known from streams in the upper portion of the Aiuruoca River drainage, an affluent of the Grande River, upper Paraná River basin, in the northern slope of Serra da Mantiqueira, southeastern Brazil (Figure 8).

#### 4.1.11 Ecological notes

**FIGURE 9:**
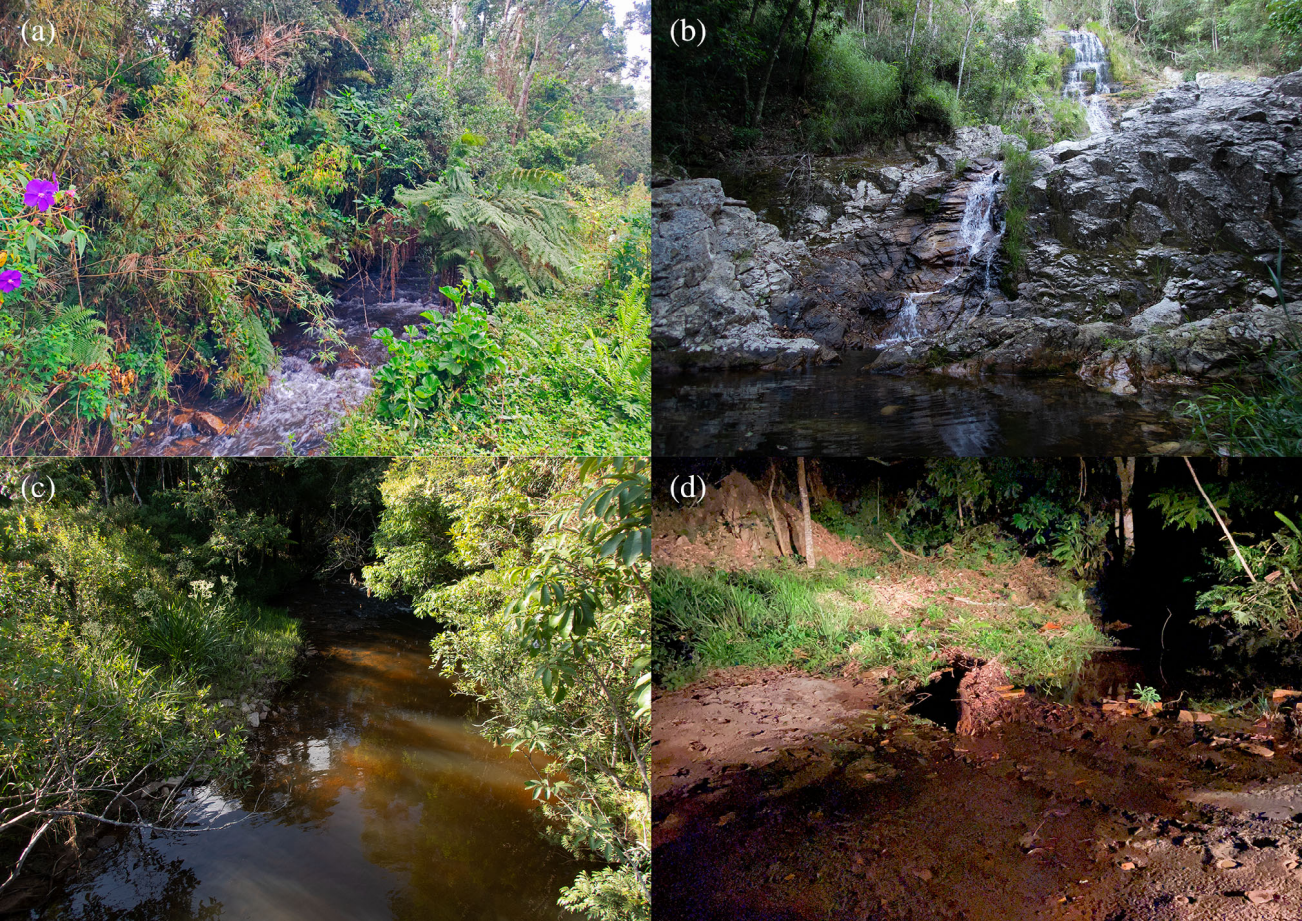
Type localities and habitats of the new *Pareiorhina* species. (a) Córrego do Quilombo, affluent to Aiuruoca River, type locality of *Pareiorhina aiuruoca* **sp. n**. (b) Cachoeira do Glicério, unnamed stream affluent to Ribeirão Itaci, type locality of *Pareiorhina isabelae* **sp. n**. (c) Ribeirão do Lavarejo, affluent to Rio Ingaí, type locality of *Pareiorhina mystica* **sp. n**. (d) Unnamed stream affluent to Ribeirão do Turvo, type locality of *Pareiorhina sofiae* **sp. n**.

*P. aiuruoca* inhabits narrow (<5 m wide) and shallow streams (<70 cm deep) in the Aiuruoca River basin, above 1000 m a.s.l., with high riparian cover in stretches of rapids with strong current and clear, cold and highly oxygenated waters. The type locality in Córrego do Quilombo is situated between two environmentally protected areas: the Itatiaia National Park and the Serra do Papagaio State Park, presenting a significant cover of native Atlantic Forest (Figure 9a). *P. aiuruoca* is mainly captured in the central portion of the streams, associated with high water current and cobbles.

#### 4.1.12 Conservation status

*P. aiuruoca* has an EOO estimated as 604 km^2^ (criterion B1), corresponding to the upper portion of the Aiuruoca River drainage. The species is known from eight streams in this drainage. Although most of the localities present significant riverine preservation, some are more prone to habitat quality loss, mainly due to deforestation and river siltation. In 2023, we sampled one of the sites where the species was registered by Casarim et al. (2012); the riparian vegetation was thin, and the stream was heavily silted, with no specimens of the new species captured. We interpret that although this species is abundant in pristine habitat conditions, it is sensitive to changes in habitat quality, especially river siltation. Additionally, we raise attention to the vulnerability of the species, considering the urban expansion around Itatiaia National Park and Serra do Papagaio State Park. However, because there is no estimate of events imminently threatening its population, we recommend *P. aiuruoca* to be listed as least concern (LC).

#### 4.1.13 Etymology

The specific epithet ‘aiuruoca’ is given in reference to the Aiuruoca River drainage in which the new species inhabits. It is a noun in apposition.

### 4.2 ***P. isabelae*, sp. n**. Azevedo‐Santos, Uzeda, Katz, Costa, Langeani & Ottoni

**FIGURE 10:**
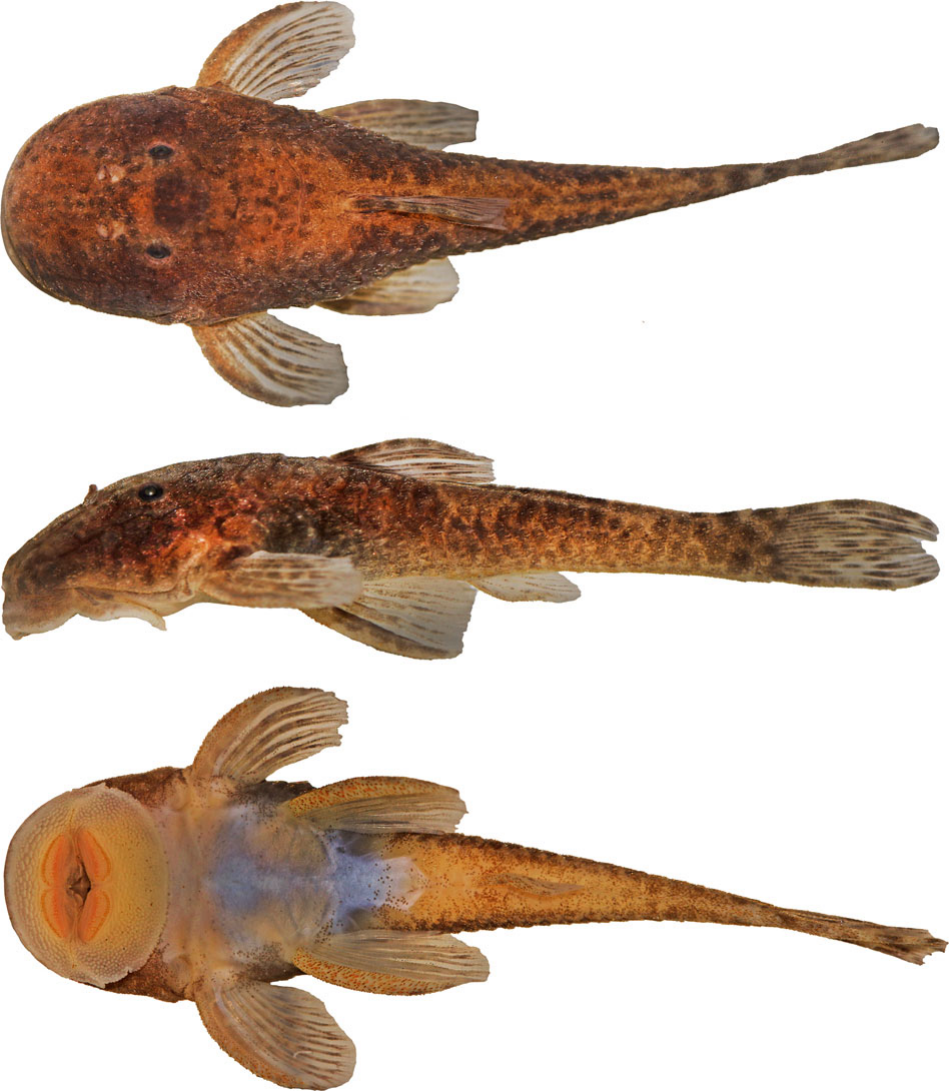
*Pareiorhina isabelae* **sp. n**., holotype, CI‐UFLA 2360, 36.4 mm standard length (SL), male, Brazil, Minas Gerais, Carmo do Rio Claro, unnamed stream affluent to Ribeirão Itaci. Dorsal, lateral and ventral views.

urn:lsid:zoobank.org:act:A93C6BD0‐E343‐45AB‐8898‐ED0A2A3DAE50

(Figures 7c,d and 10; Table 3)

‘*P. hyptiorhachis’* – Azevedo‐Santos et al., 2019: p. 7, Figure 5f [ichthyofauna inventory]

‘*Pareiorhina* sp.’ – Deprá et al., 2022: p. 337 [sympatry with *Heptapterus carmelitanorum* Azevedo‐Santos, Deprá, Aguilera, Faustino‐Fuster & Katz 2022].

‘*P. carrancas’* – Dagosta et al., 2024: p. 41, fig. 21E [*p.p*., distribution map].

#### 4.2.1 Holotype

CI‐UFLA 2360, 36.4 mm SL, male, ‘Riacho do Glicério’, downstream from Cachoeira do Glicério, affluent to Ribeirão Itaci, Sapucaí River drainage, Grande River, upper Paraná basin. Division between Carmo do Rio Claro and Ilicínea, Minas Gerais, Brazil, 20°54′58”S, 45°56′22”W, 832 m a.s.l., 02 June 2024, P.L.C. Uzeda and V.M. Azevedo‐Santos.

#### 4.2.2 Paratypes

All from Ribeirão Itaci microbasin, Minas Gerais, Brazil. **Riacho do Glicério:** CI‐UFLA 1695, 11 specimens, 5 males (28.6–35.7 mm SL), 6 females (31.2–36.2 mm SL), collected with holotype. CI‐UFLA 2333, 4 c&s specimens, 26.2–30.7 mm SL, collected with holotype. DZSJRP 23408, 6 specimens, 4 males (35.0–37.1 mm SL; 1 c&s, 35.6 mm SL), 2 females (32.5–34.0 mm SL; 1, 32.5 mm SL c&s), collected with holotype. MZUFV 13605, 4 specimens, 1 female (34.2 mm SL), 3 males (34.5–39.4 mm SL), collected with holotype. UFRJ 12831, 2 DNA, unsexed, 20.5–29.3 mm SL, 31 October 2023, A. Katz and V.M. Azevedo‐Santos. UFRJ 12855, 5 specimens, 1 male, 1 female, 3 unsexed, 26.5–39.4 mm SL, 31 October 2023, A.M. Katz and V.M. Azevedo‐Santos. UFRJ 13649, 3 DNA, unsexed, 26.4–30.5 mm SL, collected with holotype. UFRJ 14085, 7 specimens, 5 females, 2 males, 31.8–39.8 mm SL, collected with holotype. **Unnamed stream:** CI‐UFLA 1696, 10 specimens, 4 males (30.3–38.6 mm SL), 3 females (30.4–32.9 mm SL), 3 unsexed, unmeasured. Municipality of Carmo do Rio Claro at 20°55′20″S 45°58′4″W, 22 April 2023, V.M. Azevedo‐Santos et al. **Córrego Bonito:** CI‐UFLA 2416, 7 specimens, 4 females (27.7–31.9 mm SL), 2 males (26.1–28.3 mm SL), 1 unsexed (21.3 mm SL). Municipality of Carmo do Rio Claro at 20°54′40″S 45°59′22″W, 01 June 2024, P.L.C. Uzeda and V.M. Azevedo‐Santos. UFRJ 14213, DNA, 2 specimens, 22.0–25.0 mm SL, 01 June 2024, P.L.C. Uzeda and V.M. Azevedo‐Santos.

#### 4.2.3 Diagnosis

No autapomorphic states were found to diagnose *P. isabelae*; however, this species differs from *P. rudolphi*, *P. brachyrhyncha*, *P. cepta*, *P. pelicicei* and *P. rosai* by the presence of a postdorsal ridge (vs. postdorsal ridge absent) and, except for *P. rudolphi*, by having unicuspid teeth (vs. bicuspid teeth). Among congeners with a postdorsal ridge, *P. isabelae* differs from *P. carrancas* by the absence of keels on dorsal and mid‐dorsal series of plates (Figure 4a) (vs. presence, Figure 4b), by having the dorsal profile of the head rounded (vs. elliptical) and by having more premaxillary (47–73, modally 57 vs. 35–43, modally 42) and dentary (42–78, modally 53 vs. 37–43, modally 37) teeth; and from *P. hyptiorhachis* by having the snout tip naked (vs. covered with odontodes), more premaxillary (47–73, modally 57 vs. 22–44, modally 38) teeth, a single fenestra on the anterior portion of pelvic girdle (vs. two fenestrae, Figure 5e) and higher number of vertebrae (31 vs. 29).

Among the new congeners with a postdorsal ridge, *P. isabelae* can be distinguished from *P. aiuruoca* by the absence of a cluster of abdominal platelets between the pelvic fins (vs. presence), absence of dark spots over dorsal and lateral surfaces of head, body and fins (vs. spots present), by having the posterior margin of lower lip fringed (Figure 6b) (vs. smooth, Figure 6a), a single fenestra on the anterior portion of pelvic girdle (Figure 5b) (vs. two fenestrae, Figure 5a) and 31 total vertebrae (vs. 30); from *P. mystica* **sp. n**. by having the snout profile rounded (vs. elliptical), a single fenestra on the anterior portion of pelvic girdle (vs. two fenestrae, Figure 5c) and 31 total vertebrae (vs. 30); and from *P. sofiae* **sp. n**. by the tip of pectoral‐fin spine reaching the second third of dorsal‐fin base when adpressed (vs. reaching up to the base of dorsal‐fin spine, normally reaching nuchal plate), by having the snout tip naked (vs. snout tip covered with platelets), five infraorbitals (vs. four), by normally having higher counts of postdorsal ridge plates (11–16, modally 12 vs. 8–11, modally 9) and by having 31 total vertebrae (vs. 29).

#### 4.2.4 Molecular diagnosis (CBB)

*P. isabelae* is diagnosed molecularly by a combination of three nucleotide substitutions (listed below):

COI 300 (G → A), COI 381 (A → G), COI 417 (A → G).

#### 4.2.5 Description

Morphometric data and counts are presented in Table 3. Overall body shape and colouration in dorsal, lateral and ventral views are shown in Figures 7c,d and 10. Snout tip is illustrated with a small elliptical naked area. Nares located on the first third of HL. Eyes small, dorsolaterally located, slightly posterior to half of HL; eye diameter slightly larger than nares. Iris diverticulum present but poorly developed. Interorbital region about thrice the eye diameter. Parieto‐supraoccipital higher than sphenotic, posteriorly bordered by four plates. Cheeks with slightly over half the head depth; odontodes slightly hypertrophied in mature males. Mouth broad and sucker shaped. Lips broad and covered with small rounded papillae; posterior margin of lower lip fringed, with small digitiform papillae extending beyond margin of lip. Maxillary barbel short and adnate to lower lip, with short free tip. Ventral surface of head smooth, with the exception of lateral regions of cheek plate. Mandibular rami large, covered with small rounded papillae immediately behind teeth series. Dentaries angled towards each other about 160°. Teeth unicuspid, with elongated, bronze‐coloured crown; 47–73 premaxillary teeth and 42–78 dentary teeth.

Body covered by dermal plates, with the exception of ventral regions of head, abdomen, region of overlying opening of swim‐bladder capsule and around the insertions of pectoral, pelvic, dorsal and anal fins. Presence of platelets covered with odontodes clustered over lateral portion of pectoral girdle, posterior to branchiostegal membrane. Dermal plates covered with odontodes, being diffuse in juveniles; odontodes more pronounced on posterior margin of each plate. Series above lateral line roughly triangular, ventral series vertically elongated. Dorsal series with 24–26 plates; mid‐dorsal series truncated with 13–19, ending slightly posterior to dorsal‐fin base; median series with 24–27 plates; mid‐ventral series truncated with 15–20 plates, ending approximately at half the length of caudal peduncle; ventral series with 21–23 plates. Lateral line incomplete, ending at two to four posteriormost median plates. Dorsal surface of caudal peduncle with one series of 11–16 juxtaposed azygous plates, from tip of adpressed dorsal fin to procurrent caudal‐fin rays.

Dorsal fin ii,7, originating in vertical through anterior pelvic‐fin rays, reaching vertical through median anal‐fin rays when adpressed. Dorsal‐fin basal radials lying above neural spines of the 7th–14th vertebrae. Dorsal‐fin spinelet roughly rectangular, dorsal‐fin locking mechanism not functional. Pectoral fin i,6, reaching first half of pelvic fin and first half of dorsal‐fin base when adpressed. Unbranched ray curved inwards, with ventral surface covered with depressed and pointed odontodes, inward directed. Pelvic fin i,5, originating slightly anterior through dorsal‐fin origin in vertical; posterior margin falling short of anal‐fin origin in females and surpassing anal‐fin origin in males. Unbranched ray slightly curved inwards, with ventral surface plain and covered with pointed and flattened odontodes, inward oriented. Anal fin i,5, originating below posterior dorsal‐fin rays, reaching first third of caudal peduncle length when adpressed. Anal‐fin basal radials lying under haemal spines of the 14th–18th vertebrae. Caudal fin bilobed with 16 principal rays; upper lobe with I + 7 and lower lobe with 7 + I. Two dorsal procurrent caudal‐fin rays, first ray supported by epural and second ray supported by upper hypural plate. Two ventral caudal‐fin rays, both supported by the haemal spine of the 30th vertebral centrum. Total vertebrae 31; 13 precaudal e 18 caudal. Seven pleural ribs.

Supraorbital canal with four pores; pore s1 located near to anteromesial margin of nare; pore s3 located near to posteromesial margin of nare; pore s6 + s6 located at the middle of interorbital region; pore s8 located posteromesially to eye, slightly posterior to posterior orbital margin in vertical. Infraorbital canal with six pores; pore io1 located between first prerostral plate and anterior margin of first infraorbital; pore io2 located between first and second infraorbitals; pore io3 located between second and third infraorbitals; pore io4 located between third and fourth infraorbitals; pore io5 located between fourth and fifth infraorbitals; pore io6 located between superior margin of fifth infraorbital, inferior margin of sphenotic and anterior margin of compound pterotic. Preoperculomandibular canal with four pores; pore pm1 located between posterior margin of cheek plate and anterior margin of subocular cheek plate; pore pm2 located in lateral region of cheek plate; pore pm3 located between dorsal margin of cheek plate and ventral margin of preopercle; pore pm4 located between preopercle and compound pterotic. Two postotic pores; pore po2 located above branchial slit, and pore po3 located on lower half of compound pterotic.

#### 4.2.6 Colouration

Large specimens with general colour pattern light brown with dusky marks in life; all fins with light brown with irregular and inconspicuous dark bars (Figure 7c). A juvenile specimen photographed underwater in the field (not captured) presented a general colour pattern of body and fins light brown, with an irregular, broad and dark midlateral stripe (Figure 7d). Colouration in alcohol uniformly pale‐brown on dorsal and lateral regions of body and ventral surface light cream. Interradial membranes dim. Presence of zero to four faded dark bands on caudal‐fin rays.

#### 4.2.7 Sexual dimorphism

Mature males have a conspicuous, conical urogenital papilla just posterior to anal opening (absent in females) and a fleshy flap on dorsal surface of pelvic‐fin spine (absent in females). Pelvic fins slightly surpassing anal‐fin origin in males and not reaching the anal fin in females. Reproductive females present a strong enlargement in body width between dorsal‐fin origin and anal‐fin origin, resulting in a bulging trunk in dorsal view (enlargement absent in males).

#### 4.2.8 Distribution

*P. isabelae* is known from three unnamed streams within the Ribeirão Itaci microbasin, Sapucaí River drainage, Grande River, upper Paraná River basin (Figure 8).

#### 4.2.9 Ecological notes

*P. isabelae* occurs in three unnamed streams that have their watercourse originating from the Chapadão mountain (see also Deprá et al., 2022). Both streams have similar habitat structure, such as clear waters, shallow depth and rocky bottoms. At the type locality, a large number of specimens were found immediately below the largest waterfall (Figure 9b). During different expeditions in sections below the waterfall, a relatively low number of specimens were captured, and all of them were juvenile. Therefore, we hypothesize that a small migrating behaviour or an aggregated distribution pattern near waterfalls (or both) may explain the largest occurrence of the species in this section of the stream. *P. isabelae* shares its type locality with the strictly distributed *H. carmelitanorum* and *Trichomycterus adautoleitei* Costa et al., 2023 (Costa et al., 2023; Deprá et al., 2022). The description of *P. isabelae* makes the ‘Riacho do Glicério’ a potential type locality hotspot (sensu Azevedo‐Santos & Ottoni, 2025) for endemic ichthyofauna in middle Grande River basin.

#### 4.2.10 Conservation status

*P. isabelae* has an EOO estimated as 151 km^2^ (criterion B1). The species is currently known from only three streams within the Ribeirão Itaci microbasin, which flow into a section of the Sapucaí River now flooded by the Furnas Reservoir, making its tributaries fragmented (subcriterion a). Although this species has a restricted distribution, the localities where *P. isabelae* was captured presented relatively preserved habitat conditions and water quality, and there is no observation nor estimate of a decline in the EOO. Therefore, according to the IUCN criteria, we suggest *P. isabelae* to be listed as LC.

#### 4.2.11 Etymology

The specific epithet ‘isabelae’ honours Isabel Cristina de Azevedo Santos, mother of VMA‐S, who first introduced him to fishing. Isabel Cristina provided essential logistical support during the collection of this and other recently described fish species in the municipalities of Carmo do Rio Claro and Conceição da Aparecida, Minas Gerais State, Brazil. It is a noun in the genitive case.

### 4.3 ***P. mystica*, sp. n**. Uzeda, Katz, Ottoni, Costa, Langeani & Azevedo‐Santos

**FIGURE 11:**
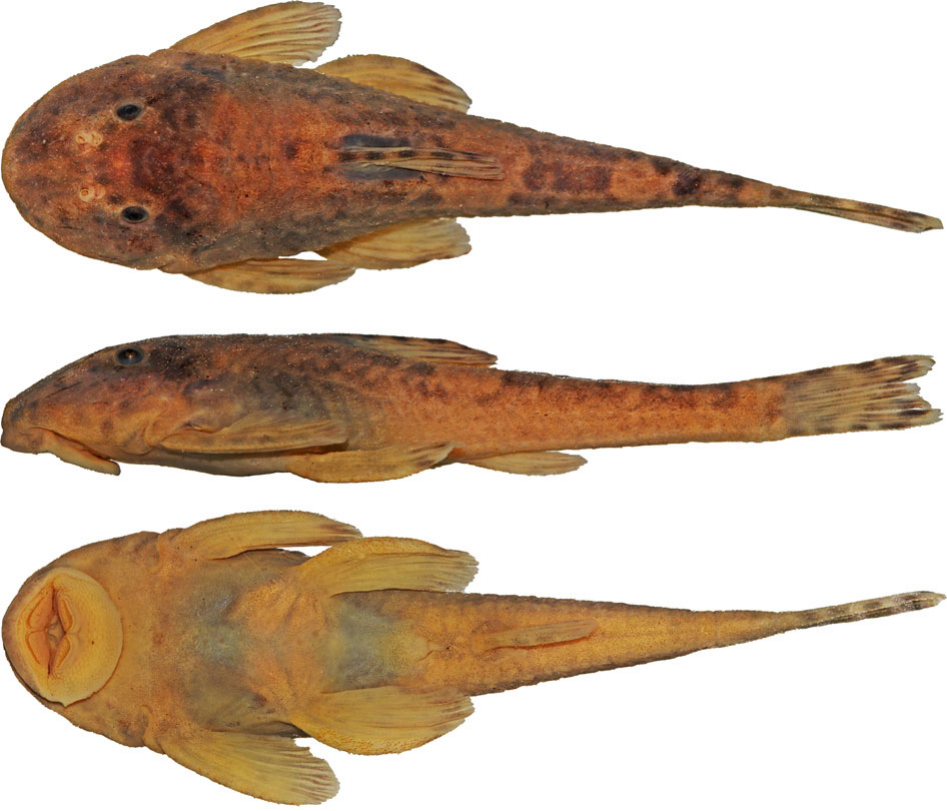
*Pareiorhina mystica* **sp. n**., holotype, CI‐UFLA 3758, 42.9 mm standard length (SL), male, Brazil, Minas Gerais, São Thomé das Letras, Ribeirão do Lavarejo. Dorsal, lateral and ventral views.

urn:lsid:zoobank.org:act:C0A242BC‐1EDC‐43 BC‐A514‐5DDDC8515ACB

(Figures 7e,f and 11; Table 4)

**TABLE 4:** Morphometric and meristic data of *Pareiorhina mystica* **sp. n**. and *Pareiorhina sofiae* **sp. n**.

Species	*Pareiorhina mystica*, sp. nov. (*n* = 21)				*Pareiorhina sofiae*, sp. nov. (*n* = 9)			
	Holotype	Range	Mean	SD	Holotype	Range	Mean	SD
Standard length	42.9	30.9–42.9	37.4	3.0	34.5	29.2–34.5	31.2	2.0
Percentage of standard length								
Head length	13.2	28.8–32.6	30.8	1.1	12.1	30.8–35.2	32.6	1.4
Predorsal length	17.2	39.3–43.0	41.3	0.9	16.4	42.3–47.5	43.8	1.6
Pre‐anal length	25.4	55.5–60.3	58.5	1.4	20.8	56.9–60.2	58.6	0.9
Dorsal‐fin spine length	8.8	17.9–22.3	20.3	1.0	6.7	18.1–22.2	20.7	1.4
Dorsal‐fin base length	6.1	12.8–16.2	14.3	0.9	5.3	12.8–15.4	14.6	0.9
Anal‐fin spine length	6.3	13.6–16.0	15.0	0.6	5.4	11.3–16.7	15.6	1.7
Pectoral‐fin spine length	8.4	17.1–20.7	19.1	0.9	5.8	16.0–18.8	18.0	0.9
Lower caudal‐fin ray length	9.5	20.5–26.2	23.0	1.5	7.8	22.6–26.9	24.6	1.6
Trunk length	6.9	13.8–17.5	15.4	0.8	4.6	12.8–17.0	14.5	1.4
Abdominal length	10.0	20.8–25.3	23.6	1.2	7.9	22.7–24.5	23.5	0.7
Pelvic‐fin spine length	9.0	15.8–23.1	20.2	1.8	7.6	20.3–25.2	22.3	1.6
Cleithral width	12.2	28.0–31.4	29.5	0.9	10.4	29.4–31.7	30.6	0.6
Body depth at dorsal‐fin origin	7.3	16.3–20.8	18.8	1.2	6.3	17.1–20.5	18.3	1.0
Body width at dorsal‐fin origin	9.0	20.9–26.0	23.8	1.4	7.6	21.1–27.7	23.5	1.9
Body width at anal‐fin origin	5.3	11.6–13.3	12.6	0.6	4.6	12.4–13.7	13.1	0.5
Caudal peduncle depth	3.2	7.3–9.9	8.5	0.6	2.7	7.8–8.8	8.2	0.3
Percentage of head length								
Head width	11.6	87.6–99.0	93.5	3.3	10.5	86.7–99.6	94.5	3.6
Snout length	7.4	55.2–62.0	58.0	1.7	7.5	45.2–61.8	58.0	5.0
Orbital diameter	1.7	12.5–15.5	13.9	0.7	1.4	11.8–14.7	12.9	1.1
Interorbital width	4.5	33.3–47.9	35.9	3.2	4.1	33.8–38.2	36.7	1.4
Head depth	6.8	49.9–69.5	56.8	4.9	6.0	49.5–60.7	52.9	3.3
Suborbital depth	4.5	31.0–38.8	34.4	2.2	4.1	32.0–36.3	34.4	1.3
Mandibular ramus	2.1	12.7–18.4	16.0	1.6	2.4	18.9–22.5	20.7	1.2
Counts			Mode				Mode	
Dorsal plates	26	24–27	26	1.0	25	24–26	25	0.8
Mid‐dorsal plates	19	14–21	18	1.9	19	16–19	19	1.1
Median plates	27	24–28	26	1.1	27	24–27	27	1.3
Mid‐ventral plates	21	17–22	20	1.4	20	18–22	19	1.1
Ventral plates	21	20–23	23	0.9	21	21–22	22	0.5
Plates between dorsal and caudal fins	16	15–17	16	0.8	14	14–16	15	0.8
Plates between anal and caudal fins	13	11–14	13	0.8	12	12–13	12	0.4
Plates at dorsal‐fin base	5	4–6	5	0.6	5	5–5	5	0.0
Plates at anal‐fin base	3	2–5	2	0.8	2	2–3	3	0.5
Postdorsal ridge plates	13	5–14	11	2.0	11	8–11	9	1.1
Dorsal‐fin branched rays	6	6–8	7	0.3	7	7–7	7	0.0
Pectoral‐fin branched rays	6	6–6	6	0.0	6	6–6	6	0.0
Pelvic‐fin branched rays	5	5–5	5	0.0	5	5–5	5	0.0
Anal‐fin branched rays	5	5–5	5	0.0	5	5–5	5	0.0
Caudal‐fin branched rays	14	14–14	14	0.0	14	14–14	14	0.0
Premaxillary teeth	33	33–55	47	5.5	44	39–50	44	4.1
Dentary teeth	31	28–56	47	7.7	41	38–54	41	5.6

Abbreviation: SD, standard deviation.

‘*P. carrancas*’ – Pompeu et al. (2009): 663 (Table 2).

‘*P. carrancas*’ – Silva‐Sene et al., 2024: p. 4 [*p.p*., ichthyofauna inventory]

‘*P. carrancas*’ – Dagosta et al., 2024: p. 41, fig. 21E [*p.p*., distribution map]

#### 4.3.1 Holotype

CI‐UFLA 3758, 42.9 mm SL, male, Ribeirão do Lavarejo, affluent to Capivari River drainage, Grande River, upper Paraná River basin. Municipality of São Thomé das Letras, Minas Gerais, Brazil, 21°37′53″S 44°53′27″W, 1070 m a.s.l., 01 December 2023, A.M. Katz, P.J. Vilardo and V.M. Azevedo‐Santos.

#### 4.3.2 Paratypes

All from Capivari River drainage, Minas Gerais, Brazil. **Ribeirão do Lavarejo:** CI‐UFLA 3759, 13 specimens, 7 females (36.6–40.8 mm SL), 2 males (36.3–42.6 mm SL), 4 unsexed (30.9–31.5 mm SL), collected with holotype. UFRJ 14044, DNA, 2 specimens, 1 female (33.4 mm SL), 1 male (37.5 mm SL), collected with holotype. UFRJ 14532, 12 specimens, 8 females, 4 males, 38.4–44.7 mm SL, collected with holotype. **Córrego da Bela Cruz:** UFRJ 14046, DNA, 2 females, 21.7–28.9 mm SL. Municipality of São Thomé das Letras at 21°37′52″S 44°51′38″W, 994 m a.s.l., 01 December 2023, A.M. Katz, P.J. Vilardo and V.M. Azevedo‐Santos. UFRJ 14084, 12 specimens, 28.7–35.6 mm SL, 01 December 2023, A.M. Katz, P.J. Vilardo and V.M. Azevedo‐Santos. **Ribeirão da Cachoeira:** CI‐UFLA 3760, 5 specimens, 2 females (36.4–36.4 mm SL), 3 males (36.2–39.0 mm SL). Municipality of Luminárias at 21°31′47″S 44°52′05″W, 1000 m a.s.l., 31 July 2023, P.L.C. Uzeda and L.J. Sartori. CI‐UFLA 3761, 4 c&s, 33.5–38.5 mm SL, 31 July 2023, P.L.C. Uzeda and L.J. Sartori. CICCAA 08621, 13 specimens, 9 females (26.2–34.5 mm SL), 1 male (38.5 mm SL), 3 unsexed (23.6–24.2 mm SL), 31 July 2023, P.L.C. Uzeda and L.J. Sartori. DZSJRP 25020, 8 specimens, 5 females (34.8–39.2 mm SL), 3 males (38.4–41.2 mm SL), 31 July 2023, P.L.C. Uzeda and L.J. Sartori. MZUFV 13606, 11 specimens, 10 females (27.8–35.8 mm SL), 1 male (37.8 mm SL), 31 July 2023, P.L.C. Uzeda and L.J. Sartori. UFMG‐ICT 4021, 13 females (19.8–36.6 mm SL), 31 July 2023, P.L.C. Uzeda and L.J. Sartori.

#### 4.3.3 Non‐type specimens

All from Capivari River drainage, Minas Gerais, Brazil. **Córrego Traituba:** CI‐UFLA 3768, 4 c&s males (34.6–39.2 mm SL). Municipality of Cruzília at 21°38′1″S 44°43′35″W, 1055 m a.s.l., April 2007, C.V. Gandini, L.S. dos Reis, R.C.R. Souza and P.S. Pompeu. **Córrego da Aroeira:** CI‐UFLA 3769, 22 specimens, 12 females (28.1–34.0 mm SL), 10 males (32.1–36.6 mm SL). Municipality of Cruzília at 21°37′55″S 44°49′8″W, 1010 m a.s.l., April 2007, C.V. Gandini, L.S. dos Reis, R.C.R. Souza and P.S. Pompeu. **Córrego Mata Grande:** CI‐UFLA 3770, 5 specimens, 2 females (30.2–32.0 mm SL), 3 males (28.5–36.1 mm SL). Municipality of Carrancas at 21°34′31”S 44°46′21”W, 1008 m a.s.l., April 2007, C.V. Gandini, L.S. dos Reis, R.C.R. Souza and P.S. Pompeu. **Córrego da Lavrinha:** CI‐UFLA 3771, 6 specimens, 4 females (29.7–34.5 mm SL), 2 males (31.2–33.5 mm SL). Municipality of Cruzília at 21°37′26”S 44°50′48”W, 982 m a.s.l., April 2007, C.V. Gandini, L.S. dos Reis, R.C.R. Souza and P.S. Pompeu.

#### 4.3.4 Diagnosis

No autapomorphic states were found to diagnose *P. mystica*; however, this species differs from *P. rudolphi*, *P. brachyrhyncha*, *P. cepta*, *P. pelicicei* and *P. rosai* by the presence of a postdorsal ridge (vs. postdorsal ridge absent) and, except for *P. rudolphi*, by having unicuspid teeth (vs. bicuspid teeth). Among congeners with a postdorsal ridge, *P. mystica* differs from *P. carrancas* by the absence of keels on dorsal and mid‐dorsal series of plates (Figure 4a) (vs. presence, Figure 4b), by having two fenestrae on the anterior portion of pelvic girdle (Figure 5c) (vs. a single fenestra, Figure 5f) and by having 30 total vertebrae (vs. 31); and from *P. hyptiorhachis* by having dorsal profile of the snout elliptical (vs. rounded), the tip of the snout naked (vs. covered with odontodes), by normally having a narrower head (87.6%–99.0% HL vs. 98.6%–110.2%) and by having 30 total vertebrae (vs. 29).

Among the new congeners with a postdorsal ridge, *P. mystica* can be distinguished from *P. aiuruoca* by the absence of a cluster of abdominal platelets between pelvic fins (vs. presence), absence of dark spots over dorsal and lateral surfaces of head, body and fins (vs. spots present), and by having the posterior margin of lower lip fringed (vs. smooth); from *P. isabelae* by having snout profile elliptical (vs. rounded), two fenestrae on the anterior portion of pelvic girdle (Figure 5c) (vs. a single fenestra, Figure 5b) and 30 total vertebrae (vs. 31); and from *P. sofiae* **sp. n**. by the tip of pectoral‐fin spine reaching the second third of dorsal‐fin base when adpressed (vs. reaching up to base of dorsal‐fin spine, normally reaching nuchal plate), by having snout tip naked (vs. snout tip covered with platelets), narrower mandibular ramus (12.7%–18.4% HL vs. 18.9%–22.5%), two fenestrae on the anterior portion of pelvic girdle (Figure 5c) (vs. a single fenestra, Figure 5d) and 30 total vertebrae (vs. 29).

#### 4.3.5 Remarks

*P. mystica* exhibits variability in abdominal plating. At the type locality, some specimens (including the holotype) bear abdominal platelets extending from the cleithrum to the pre‐anal area, whereas others possess only a single platelet between pelvic fins or lack abdominal platelets altogether. Given the lack of a consistent distribution pattern within *P. mystica*, we chose not to propose the abdominal plating as a diagnostic character for the species but rather to document its variation. It is also noteworthy that none of the specimens with abdominal platelets displayed the consistent plating pattern observed in *P. aiuruoca*.

#### 4.3.6 Molecular diagnosis (CBB)

*P. mystica* is diagnosed molecularly by a combination of four nucleotide substitutions (listed below):

COI 264 (A → G), COI 363 (A → G), COI 480 (G → A), COI 555 (C → A).

#### 4.3.7 Description

Morphometric data and counts for all type specimens are presented in Table 4. Overall body shape and colouration in dorsal, lateral and ventral views are shown in Figures 7e,f and 11. Snout tip is presented with a small elliptical naked area. Nares located on the first third of HL. Eyes small, dorsolaterally located, approximately at half of HL; eye diameter slightly larger than nares. Iris diverticulum present, but poorly developed. Interorbital region about four times eye diameter. Parieto‐supraoccipital higher than sphenotic, posteriorly bordered by four plates. Cheeks with slightly over half the head depth; hypertrophied odontodes absent. Lips broad and covered with minute rounded papillae; posterior margin of lower lip fringed, with minute digitiform papillae extending beyond margin of lip. Maxillary barbel short and adnate to lower lip, with short free tip. Ventral surface of head smooth, with the exception of lateral regions of cheek plate. Mandibular rami large, covered with minute rounded papillae immediately behind teeth series. Dentaries angled towards each other about 160°. Teeth unicuspid, with elongated bronze coloured crown; 33–55 premaxillary teeth and 28–56 dentary teeth.

Body covered by dermal plates, with the exception of ventral regions of head, abdomen, region of overlying opening of swim‐bladder capsule and around the insertions of pectoral, pelvic, dorsal and anal fins. Presence of platelets covered with odontodes clustered over lateral portion of pectoral girdle, posterior to branchiostegal membrane. Abdominal plating variable; plates randomly scattered from pectoral girdle to anal opening when present. Dermal plates covered with odontodes, being diffuse in juveniles; odontodes more pronounced on posterior margin of each plate. Series above lateral line roughly triangular, ventral series vertically elongated. Dorsal series with 24–27 plates; mid‐dorsal series truncated with 14–21, ending slightly posterior to dorsal‐fin base; median series with 24–28 plates; mid‐ventral series truncated with 17–22 plates, ending approximately at half the length of caudal peduncle; ventral series with 20–23 plates. Lateral line incomplete, ending at two to four posteriormost median plates. Dorsal surface of caudal peduncle with one series of 5–14 juxtaposed azygous plates, from tip of adpressed dorsal fin to procurrent caudal‐fin rays.

Dorsal fin ii,7, originating in vertical through anterior pelvic‐fin rays, reaching vertical through median anal‐fin rays when adpressed. Dorsal‐fin basal radials lying above neural spines of the 7th–14th vertebrae. Dorsal‐fin spinelet roughly rectangular, dorsal‐fin locking mechanism not functional. Pectoral fin I,6, reaching first half of pelvic fin and first half of dorsal‐fin base when adpressed. Unbranched ray curved inwards, with ventral surface covered with depressed and pointed odontodes, inward directed. Pelvic fin I,5, originating slightly anterior through dorsal‐fin origin in vertical; posterior margin falling short of anal‐fin origin in females and surpassing anal‐fin origin in males. Unbranched ray slightly curved inwards, with ventral surface plain and covered with pointed and flattened odontodes, inward oriented. Anal fin I,5, originating below posterior dorsal‐fin rays, reaching first third of caudal peduncle length when adpressed. Anal‐fin basal radials lying under haemal spines of 14th–18th vertebrae. Caudal fin bilobed with 16 principal rays; upper lobe with I + 7, lower lobe with 7 + I. Two dorsal procurrent caudal‐fin rays, first ray supported by epural and second ray supported by upper hypural plate. Two ventral caudal‐fin rays, both supported by the haemal spine of the 29th vertebral centrum. Total vertebrae 30, 14 precaudal e 16 caudal. Seven pleural ribs.

Supraorbital canal with four pores; pore s1 located near to anteromesial margin of nare; pore s3 located near to posteromesial margin of nare; pore s6 + s6 located at the middle of interorbital region; pore s8 located posteromesially to eye, slightly posterior to posterior orbital margin in vertical. Infraorbital canal with six pores; pore io1 located between first prerostral plate and anterior margin of first infraorbital; pore io2 located between first and second infraorbitals; pore io3 located between second and third infraorbitals; pore io4 located between third and fourth infraorbitals; pore io5 located between fourth and fifth infraorbitals; pore io6 located between superior margin of fifth infraorbital, inferior margin of sphenotic and anterior margin of compound pterotic.

Preoperculomandibular canal with four pores; pore pm1 located between posterior margin of cheek plate and anterior margin of subocular cheek plate; pore pm2 located in lateral region of cheek plate; pore pm3 located between dorsal margin of cheek plate and ventral margin of preopercle; pore pm4 located between preopercle and compound pterotic. Two postotic pores; pore po2 located above branchial slit, and pore po3 located on lower half of compound pterotic.

#### 4.3.8 Colouration

Dorsal colouration light to dark brown, overall dusky. Saddles on dorsum inconspicuous, absent in few specimens; located at dorsal‐fin origin, dorsal‐fin end, at half of caudal peduncle length and caudal peduncle end when present. Dark spots randomly dispersed over fin rays, occasionally forming four or five diffuse bands; interradial membranes transparent brown (Figure 7e,f). In ventral view, light‐beige colouration from mouth to end of caudal peduncle, with melanophores concentrated near plated areas. Colouration in alcohol similar to that in life, but with fainted tones. Dorsal bars and spots become less conspicuous in most specimens, and interradial membranes become dim.

#### 4.3.9 Sexual dimorphism

Mature males have a conspicuous, conical urogenital papilla just posterior to anal opening (absent in females) and a fleshy flap on dorsal surface of pelvic‐fin spine (absent in females). Pelvic fins slightly surpassing anal‐fin origin in males, and not reaching anal‐fin in females. Reproductive females present a strong enlargement in body width between dorsal‐fin origin and anal‐fin origin, resulting in a bulging trunk in dorsal view (enlargement absent in males).

#### 4.3.10 Distribution

*P. mystica* is known from streams within the Ingaí River microbasin, part of the Capivari River drainage, affluent to Grande River, upper Paraná River basin (Figure 8).

#### 4.3.11 Ecological notes

*P. mystica* inhabits second‐ to third‐order streams in the Ingaí River microbasin, a small drainage that flows through the Serra da Mantiqueira and Serra das Perdizes in southeastern Brazil. This region represents an ecotone between the Atlantic Forest and Cerrado biomes, encompassing a diverse array of plant formations. The landscape is generally characterized by grasslands at higher elevations and forested riparian zones (Figure 9c). The Ingaí River drains a quartzite formation, locally known as São Tomé Stone, a natural rock widely used in construction and landscaping in Brazil. As a result, streams in the region have crystal‐clear waters, with substrates primarily composed of stones and sand. The area's striking natural features, including tall waterfalls, attract numerous ecotourists.

#### 4.3.12 Conservation status

Although some localities inhabited by *P. mystica* have a long history of impacts by the extraction of the São Tomé Stone, this species is relatively abundant and easily captured in a broad area within the Ingaí microbasin. With an extent of occurrence estimated as 829 km^2^, several populations of *P. mystica* can be found in pristine localities, covered by native formations of Cerrado and Atlantic rainforest. Therefore, we suggest that *P. mystica* should be listed as LC.

#### 4.3.13 Etymology

The specific epithet ‘mystica’ is in reference to the cultural traditions and beliefs involving mysticism and esotericism, which are deeply embedded in the foundation and unique identity of the city of São Thomé das Letras and its surroundings, where the species occur. It is an adjective.

### 4.4 **P. sofiae, sp. n**. Azevedo‐Santos, Uzeda, Katz, Costa, Langeani & Ottoni

**FIGURE 12:**
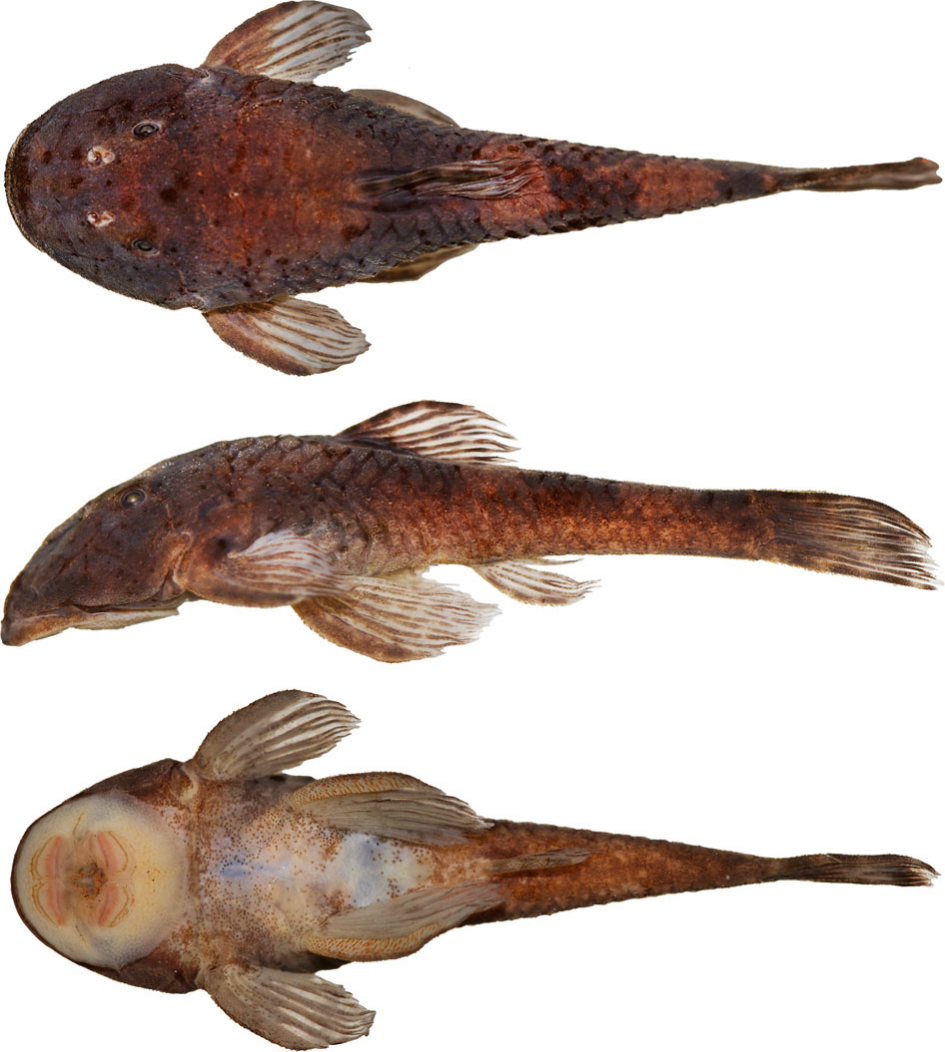
*Pareiorhina sofiae* **sp. n**., holotype, CI‐UFLA 3755, 34.5 mm SL, male, Brazil, Minas Gerais, Capitólio, unnamed stream affluent to Ribeirão do Turvo. Dorsal, lateral and ventral views.

urn:lsid:zoobank.org:act:6A2F7EDC‐4601‐41CA‐B217‐71C2D034C4CE

(Figure 12; Table 4)

#### 4.4.1 Holotype

CI‐UFLA 3755, 34.5 mm SL, male, unnamed stream affluent to Ribeirão do Turvo, Grande River, upper Paraná basin. Municipality of Capitólio, Minas Gerais, Brazil, 20°37′25″S 46°13′49″W, 802 m a.s.l., 30 May 2024, P.L.C. Uzeda and V.M. Azevedo‐Santos.

#### 4.4.2 Paratypes

All from an unnamed stream affluent to Ribeirão do Turvo. CI‐UFLA 3756, 5 specimens, 1 female (29.2 mm SL), 1 male (31.5 mm SL), 3 unsexed (25.9–26.6 mm SL), collected with holotype. CI‐UFLA 3757, 2 c&s specimens, 33.2–33.7 mm SL, collected with holotype. CICCAA 08620, 4 specimens, 1 female (30.3 mm SL), 1 male (32.7 mm SL), 2 unsexed (25.3–26.8 mm SL), collected with holotype. DZSJRP 25204, 4 specimens, 1 male (34.1 mm SL), 1 female (30.2 mm SL), 2 unsexed (25.9–27.4 mm SL), collected with holotype. UFRJ 14540, 4 specimens, 1 female (29.2 mm SL), 1 male (29.9 mm SL), 2 unsexed (24.9–27.6 mm SL), collected with holotype. UFRJ 14217, DNA, 2 specimens, 17.7–18.6 mm SL, collected with holotype.

#### 4.4.3 Diagnosis

*P. sofiae* can be autapomorphically diagnosed from all congeners by the tip of pectoral‐fin spine reaching up to the base of dorsal‐fin spine in vertical, normally reaching the nuchal plate when adpressed (vs. tip of pectoral‐fin spine reaching first half of dorsal‐fin base when adpressed in all congeners); and by having only four infraorbitals (vs. five in all other congeners). Additionally, *P. sofiae* differs from *P. rudolphi*, *P. brachyrhyncha*, *P. cepta*, *P. pelicicei* and *P. rosai* by the presence of a postdorsal ridge (vs. postdorsal ridge absent) and, except for *P. rudolphi*, by having unicuspid teeth (vs. bicuspid teeth). Among congeners with a postdorsal ridge, *P. sofiae* further differs from *P. carrancas* by the absence of keels on dorsal and mid‐dorsal series of plates (Figure 4a) (vs. presence, Figure 4b), by having snout tip covered with platelets (vs. snout tip naked), wider mandibular ramus (18.9%–22.5% HL vs. 15.4%–18.3%) and 29 total vertebrae (vs. 31); and from *P. hyptiorhachis* by presenting dorsal profile of the snout elliptical (vs. rounded), by having shorter pectoral‐fin spine (16.0%–18.8% SL vs. 20.5%–26.0%), by normally having a lower caudal peduncle (7.8%–8.8% SL vs. 8.3%–11.0%) and by having a single fenestra on anterior portion of pelvic girdle (Figure 5d) (vs. two fenestrae, Figure 5e).

Among the new congeners with a postdorsal ridge, *P. sofiae* can be further distinguished from *P. aiuruoca* by the absence of dark spots over dorsal and lateral surfaces of head, body and fins (vs. spots present), by having posterior margin of lower lip fringed (vs. smooth), snout tip covered by platelets (vs. snout tip naked), shorter pectoral‐fin spine (16.0%–18.8% SL vs. 18.9%–25.7%), a single fenestra on the anterior portion of pelvic girdle (Figure 5d) (vs. two fenestrae, Figure 5a) and 29 total vertebrae (vs. 30); from *P. isabelae* by normally having lower counts of postdorsal ridge plates (8–11, normally 9 vs. 11–16, normally 12), premaxillary teeth (39–50, modally 44 vs. 47–73, modally 57) and 29 total vertebrae (vs. 31); and from *P. mystica* by having wider mandibular ramus (18.9–22.5% HL vs. 12.7–18.4% HL), a single fenestra on the anterior portion of pelvic girdle (Figure 5d) (vs. two fenestrae, Figure 5c) and 29 total vertebrae (vs. 30).

#### 4.4.4 Molecular diagnosis (CBB)

*P. sofiae* is diagnosed molecularly by a combination of eight nucleotide substitutions (listed below):

COI 132 (A → G), COI 138 (C → T), COI 264 (A → G), COI 345 (T → C), COI 360 (G → A), COI 501 (T → C), COI 513 (A → G), COI 525 (C → T).

#### 4.4.5 Description

Morphometric data and counts are presented in Table 4. Overall body shape and colouration in dorsal, lateral and ventral views are illustrated in Figure 12.

Head deep and snout short. Snout tip covered with small platelets bearing few odontodes each. Nares located on first third of HL. Eyes small, with diameter similar to nares; dorsolaterally located, approximately on middle of HL. Iris diverticulum present and poorly developed. Interorbital region about thrice the eye diameter. Parieto‐supraoccipital higher than sphenotic, posteriorly bordered by two to four plates. Cheeks slightly above half of head depth; hypertrophied odontodes absent. Mouth broad and sucker shaped. Lips broad and covered with minute rounded papillae; posterior margin of lower lip fringed, with minute digitiform papillae extending beyond margin of lip. Maxillary barbel short and adnate to lower lip, with short free tip. Mandibular rami broad, covered with minute rounded papillae immediately behind tooth series. Dentaries angled towards each other about 160°. Teeth unicuspid, with elongated, bronze‐coloured crown; 39–50 premaxillary teeth and 38–54 dentary teeth.

Body covered with dermal plates, except for ventral surfaces of head, abdomen, the region overlying opening of swim‐bladder capsule and around bases of dorsal, pectoral, pelvic and anal fins. Presence of platelets covered with odontodes clustered over lateral portion of pectoral girdle, posterior to branchiostegal membrane. Abdominal platelets absent. Dermal plates homogeneously covered with odontodes; plates diffuse in juvenile specimens. Plate series above lateral line roughly rounded or squared, ventral series vertically elongated. Dorsal series with 24–26 plates; mid‐dorsal series truncated with 16–19 plates, ending slightly posterior to end of dorsal‐fin base; median series with 24–27 plates; mid‐ventral series truncated with 18–22 plates, ending approximately at half the length of caudal peduncle; ventral series with 21–22 plates. Lateral line incomplete, ending at two to four posteriormost median plates. Dorsal surface of caudal peduncle with one series of 8–11 juxtaposed azygous plates, from tip of adpressed dorsal fin to procurrent caudal‐fin rays.

Dorsal fin ii,7, originating at vertical through median pelvic‐fin rays, slightly surpassing anal‐fin base when adpressed. Dorsal‐fin basal radials lying above neural spines of the 7th–14th vertebrae. Dorsal‐fin spinelet roughly rectangular; locking mechanism not functional. Pectoral fin i,6, reaching first half of pelvic fin and first third of dorsal fin in vertical when adpressed; presence of inconspicuous dark spots over rays. Pelvic fin i,5, originating slightly anterior to dorsal‐fin origin in vertical; posterior margin reaching halfway between anal opening and anal‐fin origin in females and surpassing anal‐fin origin in males. Unbranched ray curved inwards, its ventral surface covered with depressed, pointed and inward directed odontodes. Anal fin i,5, originating slightly posterior to end of dorsal‐fin base, reaching about half the length of caudal peduncle when adpressed. Anal‐fin basal radials lying below haemal spines of 13th–16th vertebrae. Caudal fin bilobed, with 16 principal rays; upper lobe with I + 7 and lower lobe with 7 + I. Two dorsal and two ventral procurrent rays, respectively, supported by neural and haemal spines of the 28th vertebral centrum. Total vertebrae 29; 13 precaudal and 16 caudal. Six pleural ribs.

Supraorbital sensory canal with four pores; pore s1 located anteromesially to nare, pore s3 located posteriomesially to nare; pore s6 + s6 located in middle of interorbital region, pore s8 located posteromesially to eye, longitudinally aligned to mesial margin of nare. Infraorbital sensory canal with five or six pores; pore io1 located at the middle of inferior margin of first infraorbital; pore io2 located between first and second infraorbitals; pore io3 located between second and third infraorbitals; pore io4 located between third and fourth infraorbitals; pore io5, when present, located at the middle of fourth infraorbital and pore io6 located between superior margin of fourth infraorbital, inferior margin of sphenotic and anterior margin of compound pterotic. Preoperculomandibular canal with four pores; pore pm1 located between posterior margin of cheek plate and anterior margin of subocular cheek plate; pore pm2 located in lateral region of cheek plate; pore pm3 located between dorsal margin of cheek plate and ventral margin of preopercle; pore pm4 located between preopercle and compound pterotic. Two postotic pores; pore po2 located above branchial slit, and pore po3 located on lower half of compound pterotic.

#### 4.4.6 Colouration

Overall colouration homogeneously dark brown; spots or vermiculations absent. Presence of four light saddles on dorsum, located at dorsal‐fin origin, dorsal‐fin end, half of caudal peduncle length and caudal peduncle end. Ventral surface of body light beige, except for plated areas, and few melanophores concentrated around bases of fins. Interradial membranes of all fins hyaline; fin rays dusky‐brown pigmented, not forming bars.

#### 4.4.7 Sexual dimorphism

Mature males have a conspicuous, conical urogenital papilla just posterior to anal opening (absent in females) and a fleshy flap on dorsal surface of pelvic‐fin spine (absent in females). Pelvic fins slightly surpassing anal‐fin origin in males and not reaching anal‐fin in females. Reproductive females present a strong enlargement in body width between dorsal‐fin origin and anal‐fin origin, resulting in a bulging trunk in dorsal view (enlargement absent in males).

#### 4.4.8 Distribution

*P. sofiae* is only known from a single stream in the Ribeirão do Turvo microbasin, a small drainage affluent to Grande River at the current Furnas Reservoir, southern slope of Serra da Canastra, southeastern Brazil (Figure 8).

#### 4.4.9 Ecological notes

*P. sofiae* inhabits a steep, first‐order stream draining a valley in Serra da Canastra. The stream is intersected by an unpaved road. Due to its shallow depth (<40 cm) and width (<2 m), no bridges were constructed, and vehicles cross directly through the stream. At the river stretch where the road was constructed, the water column becomes vertically constricted and flows as a thin layer over the road surface (Figure 9d). The stream runs for a few metres across the road until it turns into a sequence of short waterfalls and runs freely to its mouth in Ribeirão do Turvo. Few specimens of *P. sofiae* were captured both upstream and downstream from the unpaved road; however, this species has never been captured in the main channel of Ribeirão do Turvo.

#### 4.4.10 Conservation status

*P. sofiae* has an EOO estimated as 118 km^2^, corresponding to the entire Ribeirão do Turvo microbasin (criterion B1). However, the species is known only from a single stream within this microbasin and was not captured in adjacent streams, either inside or outside the basin (subcriterion a). Habitat alterations caused by the rural road crossing the stream, such as impoundment upstream and siltation downstream, pose significant threats to the unique known population (subcriteria biii, biv). Therefore, and according to the IUCN criteria, we recommend *P. sofiae* to be listed as endangered (EN).

#### 4.4.11 Etymology

The specific epithet ‘*sofiae*’ is in honour of Marlene Sofia Arcifa for her contributions to the ecological knowledge and conservation of Neotropical aquatic ecosystems. It is a noun in the genitive case.

## 5 DISCUSSION

Our integrative approach proved highly effective in delimiting distinct evolutionary units within *Pareiorhina*, as each unit exhibited unique morphological profiles, corroborated by multiple molecular species delimitation methods. Key diagnostic features, such as the presence or absence of a postdorsal ridge, simple or bicuspid teeth and vertebral counts showed strong congruence with molecular species delimitation. Character states related to lip papillae have also proven informative in Neoplecostomini, such as the presence of a large fleshy bump on dentary symphysis in *Neoplecostomus doceensis* Roxo, Silva, et al., 2014, and the presence of coalesced lip papillae in *Pareiorhaphis torrenticola* Pereira et al., 2024 (Pereira et al., 2024; Roxo, Silva, et al., 2014). We introduce this character to *Pareiorhina* through *P. aiuruoca*, which consistently exhibits a smooth posterior margin of the lower lip, whereas all other species bearing a postdorsal ridge exhibit a fringed lower lip (Figure 6).

We also brought up a new character that proved to be efficient in the morphological delimitation of species in *Pareiorhina*: the morphology of the pelvic girdle. In our analysis, each species exhibited consistent morphology, particularly regarding the formation of fenestrae on the anterior portion of the pelvic girdle. As shown by Pereira and Reis (2017), in most of the Neoplecostominae (=Neoplecostomini), the anterolateral processes of the basipterygium are curved and converge mesially, resulting in paired fenestrae between the anterolateral and anteriomesial processes (Char. 191.1), as seen in *P. aiuruoca*, *P. mystica* and *P. hyptiorhachis* (Figure 5a,c,e). On the contrary, the anteromesial processes of the basipterygium are reduced in *P. isabelae*, *P. sofiae* and *P. carrancas*, resulting in a single medial fenestra (Figure 5b,d,f).

The absence of abdominal plates has historically been used to diagnose the genus *Pareiorhina* (Bockmann & Ribeiro, 2003), with *P. cepta* as the only species initially described with minute platelets scattered on the abdomen (Roxo, Silva, et al., 2012). However, we observed minute abdominal platelets or interspaced odontodes in several species, including *P. carrancas*, *P. hyptiorhachis*, *P. cepta*, *P. rosai*, *P. aiuruoca* and *P. mystica*. Additionally, all *Pareiorhina* species except *P. rudolphi* exhibit a small patch of odontode‐bearing platelets on the lateral portion of the pectoral girdle, posterior to branchiostegal membranes, suggesting that this character warrants reevaluation. Notably, *P. aiuruoca* is the only species that exhibits a consistent arrangement of large platelets between the pelvic fins, most of which support more than 10 odontodes each in adult specimens (Figure 5a). In contrast, *P. rudolphi* consistently lacks abdominal platelets from pectoral girdle to anal opening.

We obtained major congruence among the species delimitation methods tested, including distance‐, tree‐ and coalescent‐based approaches. All methods delimited species with relatively low genetic divergence, in some cases below 1% (e.g., *P. cepta* vs. *P. pelicicei*), highlighting the limitations of the arbitrary 2% threshold, which fails to account for lineage‐specific evolutionary histories. Additionally, genetic distances must be interpreted cautiously, especially when comparing recently diverged lineages. As proposed by Roxo, Zawadzki, et al. (2012), the ancestors of *P. carrancas* and *P*. sp. 1 (= *P. hyptiorhachis*) likely originated in continental drainages and expanded to coastal systems approximately 6 million years ago. This recent evolutionary history may explain the low genetic divergence observed, although all species exhibited coalescent patterns consistent with independently evolving lineages.

The polyphyly of *Pareiorhina* has been recovered in multiple molecular phylogenies based on mitochondrial and nuclear markers, as well as ultraconserved elements, indicating that *P. brachyrhyncha*, *P. carrancas* and *P. hyptiorhachis* are not closely related to *P. rudolphi* (Cramer et al., 2011; Roxo et al., 2019; Roxo, Albert, et al., 2014; Roxo, Zawadzki, et al., 2012). Although resolving the phylogenetic relationships within *Pareiorhina* is beyond the scope of our study, we recovered similar results using all valid species of the genus, which could indicate that all species allocated in *Pareiorhina* after *P. rudolphi*, including the new species described herein, likely belong to a new, undescribed genus – hereafter, ‘*Pareiorhina*’. Nevertheless, we provisionally allocate the new species in *Pareiorhina* due to their close phylogenetic relationships with the species currently valid as *P. carrancas* and *P. hyptiorhachis*, until a comprehensive, integrative revision of the genus clarifies its evolutionary history and supports formal taxonomic changes.

Within ‘*Pareiorhina*’, members of the ‘*P*.’ *carrancas* clade can be diagnosed by the presence of a postdorsal ridge, formed by 5–16 azygous plates on the dorsal surface of the caudal peduncle and the presence of simple teeth (Bockmann & Ribeiro, 2003; Silva et al., 2013), whereas the ‘*P*.’ *brachyrhyncha* clade is characterized by the absence of a postdorsal ridge and presence of bicuspid teeth (Chamon et al., 2005; Roxo, Silva, et al., 2012; Silva et al., 2016). In turn, *P. rudolphi* is distinguished by a completely naked abdomen, caudal peduncle distinctly flat dorsally and ventrally and unicuspid teeth (Pereira & Reis, 2017).

The ‘*P*.’ *carrancas* clade is composed of two subclades: one including ‘*P*.’ *carrancas*, ‘*P*.’ *aiuruoca*, ‘*P*.’ *hyptiorhachis* and ‘*P*.’ *mystica*, and another comprising ‘*P*.’ *isabelae* and ‘*P*.’ *sofiae*. This arrangement might be reflecting biogeographic patterns, as ‘*P*.’ *isabelae* and ‘*P*.’ *sofiae* occur in the Serra da Canastra ecoregion (middle Grande River drainage), whereas the former inhabit the Serra da Mantiqueira ecoregion in streams draining the upper Grande River and adjacent basins, including ‘*P*.’ *hyptiorhachis* in the headwaters of the Paraíba do Sul River basin (Azevedo‐Santos et al., 2019; Casarim et al., 2012; Pompeu et al., 2009; Silva et al., 2013). The ‘*P*.’ *carrancas* clade is currently known from the Grande River (upper Paraná) and Paraíba do Sul basins. Unlike the ‘*P*.’ *brachyrhyncha* clade, it has not yet been recorded from the São Francisco basin, though it is too early to determine whether this reflects sampling gaps or a true absence.

To date, three of the newly described species (viz. ‘*P*.’ *aiuruoca*, *‘P.’ isabelae* and *‘P'. mystica*) had been previously cited as *P. carrancas* (Dagosta et al., 2024; Silva‐Sene et al., 2024). The dismantling of ‘*P. carrancas*’ in the Grande River reshapes the ecological and conservation scenario for small loricariids in the region, as at least four narrowly distributed evolutionary units were hidden under this name. Similar patterns of narrowly distributed species have been observed with other fish groups in the middle and upper portions of the Grande River, such as trichomycterines (Costa et al., 2023; Costa et al., 2025; Costa & Katz, 2021), the enigmatic *H. carmelitanorum* (Azevedo‐Santos et al., 2019; Deprá et al., 2022), the neoplecostomines *Neoplecostomus altimontanus* Uzeda et al. 2024 and *Neoplecostomus sapucai* Andrade et al. 2024 (Uzeda et al., 2024), and even the endangered, monotypic tetra *Lophiobrycon weitzmani* Castro et al., 2003 (Castro et al., 2003).

The description of ‘*P*.’ *aiuruoca*, *‘P.’ isabelae*, *‘P*.*’ mystica* and *‘P.’ sofiae* triples the known diversity of the genus in the upper Paraná River basin, now comprising six species. These findings reinforce the presence of cryptic diversity within Loricariidae and highlight biogeographic patterns of endemism among small fish species inhabiting headwater streams in southeastern Brazil. The integration of molecular and morphological data remains essential to uncover and interpret this hidden diversity and its underlying patterns.

**COMPARATIVE MATERIAL**

**All from southeastern Brazil**. Pareiorhina carrancas: Córrego Debaixo da Serra, affluent to Grande River, upper Paraná River basin: CI‐UFLA 1510, 15 specimens, 27.7–40.9 mm SL; CI‐UFLA 3762, 2 c&s specimens, 34.8–38.9 mm SL; UFRJ 10149, 18 specimens, 14.3–42.8 mm SL; UFRJ 10248, 5 c&s specimens, 18.0–36.2 mm SL. Pareiorhina hyptiorhachis: Ribeirão Fernandes, affluent to Pomba River, Paraíba do Sul River basin: LBP 12248, 1 c&s, paratype. Tinguá River, affluent to Pomba River, Paraíba do Sul River basin: LISDEBE 7875, 6 specimens + 2 c&s, 28.1–38.0 mm SL. Pareiorhina cepta: Córrego Lavapés, affluent to Samburá River, São Francisco River basin: CI‐UFLA 2413, 20 specimens, 21.7–40.5 mm SL; UFRJ 12856, 2 specimens, 39.4–49.0 mm SL. Pareiorhina pelicicei: Córrego Tamborete, affluent to Grande River, upper Paraná River basin: CI‐UFLA 3772, 5 specimens, 34.5–40.0 mm SL; UFRJ 12854, 9 specimens, 26.7–45.6 mm SL. Pareiorhina rosai: Córrego da Mutuca, affluent to das Velhas River, São Francisco River basin: CI‐UFLA 0129, 29 specimens, 18.5–36.0 mm SL; UFRJ 12256, 5 specimens, 26.3–31.1 mm SL. Pareiorhina rudolphi: Ribeirão Benfica, Paraíba do Sul River basin: UFRJ 10327, 5 specimens, 49.0–54.5 mm SL; UFRJ 10940, 3 c&s specimens, 50.0–54.0 mm SL; UFRJ, 12361, 28 specimens, 28.5–51.2 mm SL; UFRJ 12367, 1 c&s specimen, 53.0 mm SL; UFRJ 12461, 3 c&s specimens, 52.0–55.0 mm SL; UFRJ 13260, 3 c&s specimens, 48.9–60.4 mm SL.

## 6 AUTHOR CONTRIBUTIONS

**Pedro L. C. Uzeda:** conceptualization, methodology, software, validation, formal analysis, investigation, data curation, writing ‐ original draft, visualization, supervision. **Luana J. Sartori:** methodology, investigation, writing ‐ review and editing, visualization. **Axel M. Katz:** methodology, investigation, software, formal analysis, investigation, resources, data curation, writing ‐ review and editing. **Felipe P. Ottoni:** methodology, software, validation, investigation, resources, data curation, writing ‐ review and editing, supervision, project administration, funding acquisition. **Wilson J. E. M. Costa:** methodology, validation, resources, data curation, writing ‐ review and editing. **Francisco Langeani:** methodology, validation, formal analysis, investigation, data curation, writing ‐ review and editing, supervision. **Valter M. Azevedo‐Santos:** conceptualization, investigation, data curation, writing ‐ review and editing, supervision, project administration.

## FUNDING INFORMATION

This study was supported by Conselho Nacional de Desenvolvimento Científico e Tecnológico (CNPq; grant 304755/2020–6 to Wilson J.E.M. Costa, grant 306490/2024‐2 to Felipe P. Ottoni and grant 313769/2023–0 to Francisco Langeani), Fundação de Amparo à Pesquisa do Estado de Minas Gerais (Project BIOTAMINAS/FAPEMIG, and grants 15385 and 15381 to Pedro L.C. Uzeda and Luana J. Sartori, respectively), Fundação Carlos Chagas Filho de Amparo à Pesquisa do Estado do Rio de Janeiro (FAPERJ; grant E‐26/201.213/2021 to Wilson J.E.M. Costa and E‐26/202.005/2020 to Axel M. Katz) and Mohamed bin Zayed Species Conservation Fund (MBZ Project number 232531963 to Felipe P. Ottoni). This study was also supported by CAPES (Coordenação de Aperfeiçoamento de Pessoal de Nível Superior), Finance Code 001 through the Programa de Pós‐Graduação em: Biodiversidade e Biologia Evolutiva/UFRJ and Genética/UFRJ.

## CONFLICT OF INTEREST STATEMENT

The authors declare no conflict of interest.

## Supporting information


**TABLE S1.** List of specimens used in our molecular analyses. Letters in parenthesis indicate the river basins as PS (Paraíba do Sul), SF (São Francisco) and UP (upper Paraná). Municipalities are followed by state as MG (Minas Gerais) and SP (São Paulo).

## Data Availability

All sequences used in the present work are available in Supporting Information, Table S1.
